# Lepidopteran prolegs are novel traits, not leg homologs

**DOI:** 10.1126/sciadv.add9389

**Published:** 2023-10-12

**Authors:** Yuji Matsuoka, Suriya Narayanan Murugesan, Anupama Prakash, Antónia Monteiro

**Affiliations:** Department of Biological Sciences, National University of Singapore; 14 Science Drive, Singapore 117543 Singapore.

## Abstract

Lepidopteran larvae have both thoracic legs and abdominal prolegs, yet it is unclear whether these are serial homologs. A RNA-seq analysis with various appendages of *Bicyclus anynana* butterfly larvae indicated that the proleg transcriptome resembles the head-horn transcriptome, a novel trait in the lepidoptera, but not a thoracic leg. Under a partial segment *abdominal-A* (*abd-A*) knockout, both thoracic leg homologs (pleuropodia) and prolegs developed in the same segment, arguing that both traits are not serial homologs. Further, three of the four coxal marker genes, *Sp5*, *Sp6-9*, and *araucan*, were absent from prolegs, but two endite marker genes, *gooseberry* and *Distal-less*, were expressed in prolegs, suggesting that prolegs may be using a modular endite gene-regulatory network (GRN) for their development. We propose that larval prolegs are novel traits derived from the activation of a pre-existing modular endite GRN in the abdomen using *abd-A*, the same Hox gene that still represses legs in more lateral positions.

## Teaser

Lepidopteran prolegs are not leg homologs but appear to be atavistic endites that were present in basal hexapods.

## INTRODUCTION

**Fig. 1.**
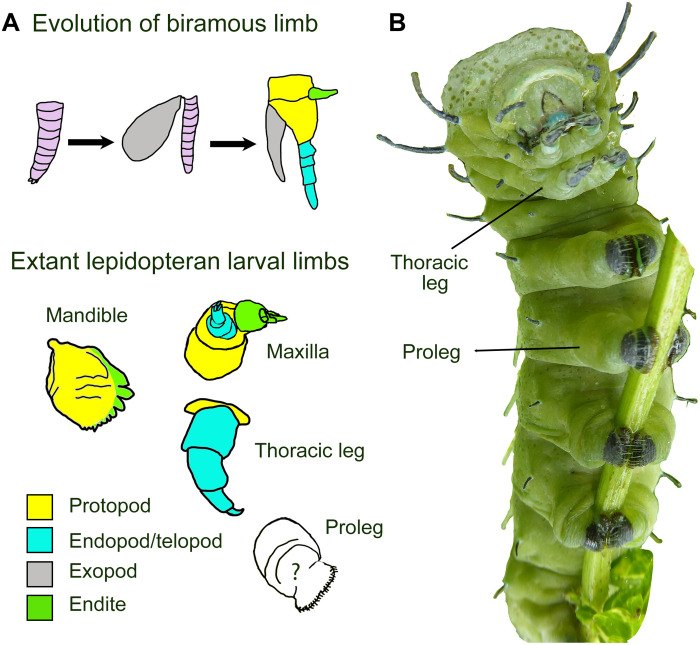
Evolution of the arthropod limb and type of lepidopteran larval limbs, including prolegs. (**A**) Evolution of the ancestral biramous arthropod limb from a monopodial limb and derived lepidopteran larval limbs, with highlighted subdivisions [inspired from (*3*)]. Some head appendages (e.g, maxilla) have a main axis (telopod) and lobes (endites) that grow from the basal part of the limb (the protopod). Thoracic legs are uniramous and end in a single claw, whereas prolegs are fleshy and have many crochets at the tip. (**B**) *Attacus atlas* caterpillar (photo by Antónia Monteiro).

Cambrian fossils show that the ancestors to all arthropods had biramous limbs, posterior to the uniramous first antennae, that diversified into all other limb types (*1*). This biramous limb was initially a simple monopodial limb as found in lobopodians (onychophorans and tardigrades) (*2*). The single limb subsequently evolved into a two-branched limb, containing the exopod and the endopod. Later, a proximal structure called the protopod differentiated (Fig. 1A), and structures called endites and exites evolved on the ventral and dorsal sides of the protopod, respectively (*1*). All insect limbs, however, including legs and mouthparts, have lost the exopod and have become uniramous, the distal segments of that single ramous now being called the telopod (Fig. 1A) (*1*). Legs have also lost endites, but these are still visible as the gnathal edge of the mandible, the lacinia and galea of the maxilla, and the glossa and paraglossa of the labium (Fig. 1A) (*3*, *4*).

In addition to changes in structure, limbs have also been evolving in number. While most adult insects have three pairs of walking legs, juveniles display tremendous variation in both thoracic and abdominal appendages, only some of which are used for walking (*5*–*7*). The origin, development, and evolution of this diversity in appendage number in insects, however, are still poorly understood.

In Lepidoptera, for instance, the developmental origin of the abdominal prolegs is still controversial. Prolegs are stubby and fleshy appendages, lacking a claw, that emerge from the ventral surface of several segments in the abdomen of most lepidoptera and that help them grasp onto surfaces (Fig. 1B). They have been hypothesized to be serial homologs to thoracic legs (*8*), completely novel traits (*9*), or modified endites of primitive abdominal appendages (*7*).

In the classical view proposed by Snodgrass (*8*), prolegs are leg serial homologs containing just the proximal coxa and subcoxal segments that make up the protopod (yellow region in Fig. 1A). Birket-Smith (*10*) supported this view by showing that the musculature and innervation of prolegs resembled those observed in thoracic legs. Later molecular studies revealed that the gene *Distal-less* (*Dll*), which patterns the telopod (blue regions in Fig. 1A), was expressed during proleg and thoracic leg development (*11*, *12*), supporting the serial homology hypothesis further, but not supporting Snodgrass’ proximal coxal identity hypothesis for prolegs. More recently, Bruce and Patel proposed that prolegs are homologous to small abdominal nubs seen in *Tribolium* embryos (*13*), consisting only of the protopod, which are incorporated into body wall before hatchling. This is a similar hypothesis to that of Snodgrass, if we assume that these nubs are not incorporated into body wall in lepidopterans and give rise to prolegs instead.

In contrast, other researchers proposed that prolegs are novel traits. Either because they originated in the Lepidoptera from ancestors without abdominal appendages (*9*) or because they develop in a more medio-ventral position relative to thoracic legs (*14*–*16*).

Last, others proposed that prolegs likely derive from endites growing from the coxopodite, which are present in basal apterygote hexapods. Members of the Diplura and Machilidae have two pairs of abdominal processes in each segment called styli (more laterally located) and eversible vesicles (more medially located) (*17*, *18*). These two traits grow out from a single region, the coxopodite, that is incorporated into body wall (*17*, *18*). Machida (*17*) proposed that the stylum is homologous to the telopod and that the eversible vesicle is homologous to a coxal endite of more primitive arthropods. Bitsch (*7*) later proposed that these coxal endites are homologous to prolegs in the lepidoptera.

Here, we set out to test these multiple hypotheses of proleg origins by (i) comparing the transcriptomes of prolegs to several other appendicular organs, (ii) manipulating Hox genes known to transform serial homologs into distinct identities, and (iii) examining the expression of genes known to be markers for specific parts of arthropod appendages. In particular, the gene *Dll* is considered a marker not only for the telopod (*19*) but also for endites (*4*, *20*), whereas a gene such as *homothorax* (*hth*) is a marker for the protopod across all arthropods (*21*). *Sp5* (*buttonhead* in *Drosophila*) and *Sp6-9* (*Sp1* in *Drosophila*) are markers for both the protopod and telopod (*22*, *23*). A pair-rule family gene, *paired*, was found to be a marker for endites in the mandible and maxilla of *Tribolium* (*4*), whereas *araucan* (*ara*) was found to be expressed in the protopod of both a crustacean and *Tribolium* (*19*), but neither gene has been examined in lepidopteran embryos.

## RESULTS

**Fig. 2.**
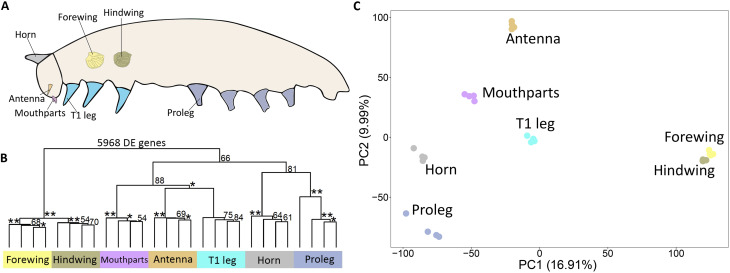
RNA-seq analysis shows the proleg transcriptome is closest to head horn. (**A**) Tissues extracted from the late larval stage for RNA sequencing. (**B**) Hierarchical clustering using 5968 DE genes from pairwise comparisons between the different tissues. **100 approximately unbiased (AU) *P* value; *90 to 99 AU *P* value. (**C**) PCA constructed using the variance-stabilizing transformation (VST) counts of 5968 DE genes. PC, principal component.

To examine how proleg and leg gene-regulatory networks (GRNs) compare to each other and to other appendages in the body, we sampled total RNA from several fifth instar larval appendages [as in (*24*)] (Fig. 2A) and sequenced their transcriptomes. We identified 5968 differentially expressed (DE) genes [log fold change (FC) ≥ |2| and *P* adjusted (*P* adj.) = 0.001] across all tissues using pairwise DE analyses. Hierarchical clustering (HC) and principal components analyses (PCAs) constructed using the DE genes showed prolegs clustered closest to head horns and formed a sister clade to legs, antennae, and mouthparts, with forewings and hindwings forming an outgroup (Fig. 2, B and C).

To examine whether proleg-specific DE genes also produced the same clustering pattern, we identified proleg-specific DE genes (logFC ≥ |1| and *P* adj. = 0.05) by comparing prolegs with abdominal regions without prolegs (fig. S1 and data S1). HC analyses using the identified 2060 proleg DE genes resulted in prolegs clustering, on their own, to a group containing all other appendages, except wings, the outgroup again in this analysis (fig. S2A). We also performed HC using 25 leg-specific genes (identified from the *Drosophila* literature), which resulted in prolegs clustering with horns, hindwings, and forewings, and separately from legs, antennae, and mouthparts, highlighting that prolegs and legs have different transcriptome profiles (fig. S2B).

When we repeated the HC with a subset of 5968 DE genes corresponding to DNA transcription factors and DNA binding proteins [Gene Ontology (GO):0003700 and GO:0008134], which are the building blocks of GRNs, prolegs, and horns clustered together, forming a sister clade to all other tissues including the wings (fig. S2C). Overall, these results suggest that at the fifth instar larval stage, the proleg and head-horn GRNs are most similar.

Previous research demonstrated that Hox genes regulate the number and type of abdominal larval appendages in several insect lineages. For instance, *Ultrabithorax* (*Ubx*), which is normally expressed in abdominal segments 1 and 2 (A1 and A2) (*25*), modifies the appendage primordia of A1 of several insects into a transient embryonic glandular organ, the pleuropodium (*26*, *27*, *28*), that secretes a chitinase to facilitate embryonic hatching (*29*). When *Ubx* was down-regulated in *Tribolium*, pleuropodia were transformed into a partial thoracic leg, suggesting that the pleuropodium is a leg homolog (*26*). Another Hox gene, *abdominal-A* (*abd-A*), expressed from the posterior of the A1 segment onward, acts as a limb repressor in these more posterior segments in both *Tribolium* and *Drosophila* via the repression of *Dll* (*26*, *30*). When *abd-A* was down-regulated, ectopic *Dll* expression and ectopic pleuropodia formed throughout the abdomen of *Tribolium*, and when both *Ubx* and *abd-A* were down-regulated, legs formed in all abdominal segments (*26*).

In Lepidoptera, *Ubx* and *abd-A* seem to have distinct effects on abdominal appendage development relative to beetles. In this lineage, pleuropodia develop in A1, no appendage develops in A2, and prolegs develop in A3 to A7 (*11*, *25*). When both *Ubx* and *abd-A* were mutated in *Bombyx mori*, thoracic legs formed in abdominal segments A1 to A7 (*31*), same as in beetles, showing that both genes repress thoracic legs. However, mutations or down-regulation of *abd-A* alone led to the loss of prolegs, showing that *abd-A* is required for proleg development (*31*–*33*). Furthermore, when *abd-A* was overexpressed in A2 (due to a regulatory mutation) where no prolegs develop, prolegs developed in A2 (*34*), but when *Ubx* was overexpressed in embryos (via a regulatory mutation), prolegs disappeared (*35*). These results indicate that *abd-A* is essential for proleg development while also repressing thoracic legs, whereas *Ubx* represses prolegs while also promoting pleuropodia in A1 in Lepidoptera.

The results above show two instances of the same Hox gene having opposite effects on appendage development in different segments of one species. This can be easily explained if thoracic legs and prolegs are different traits using separate GRNs. Each Hox gene would interact with each of the GRNs in a different way. *Ubx* could be both a modifier of the thoracic leg GRN, transforming it into pleuropodia in A1, and a repressor of the proleg GRN, preventing prolegs from developing in A2. Likewise, *abd-A* could be both a thoracic leg GRN repressor in A2 and more posterior segments and an activator of the proleg GRN in those same segments. The two-trait hypothesis can be tested under a partial segment Hox gene knockout, where the two types of traits might be able to develop side by side in the same segment (*36*).

Before testing the two-trait hypothesis, we first examined the detailed expression domains of three Hox proteins, Antennapedia (Antp), Ubx, and Abd-A, and the pleuropodia/telepod/proleg marker protein Dll in *Bicyclus anynana* and then observed how CRISPR-Cas9 disruptions of each Hox gene affected thoracic and abdominal limb development and the presence of Dll protein during embryonic development.

Typical expression domains were found for all three proteins (fig. S3). Antp proteins were observed in the thoracic segments, Ubx proteins were observed in T3 and anterior compartments of A1 and A2 segments, and Abd-A proteins were present from the posterior part of A1 to A7 and part of A8 (fig. S3, A, D, and G). These three Hox proteins were also present in the developing embryonic prolegs: Ubx in the more medial half-rim of the proleg tip, where Abd-A was absent (fig. S3C); Abd-A at the base of the proleg, (fig. S3E and F); and Antp at the periphery of the proleg tips (fig. S3, H and I). Dll was expressed at the proleg tip (fig. S3, E and F). Most of these expression domains were previously observed in a different butterfly (*11*).

**Fig. 3.**
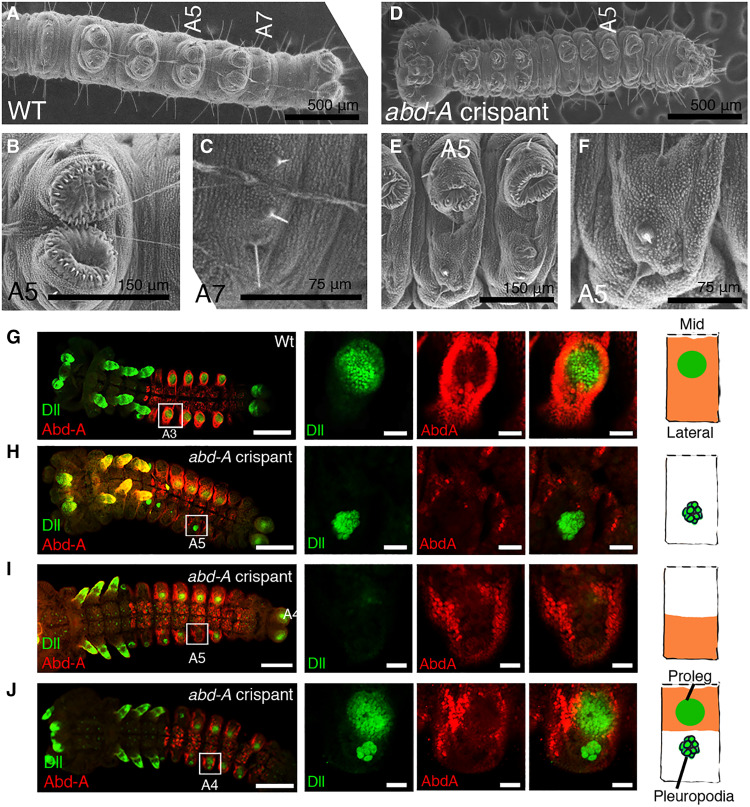
*abd-A* is necessary for proleg development and for repressing pleuropodia. (**A** to **C**) Scanning electron microscopy image of wild-type (WT) first instar larvae. Wild-type prolegs (B) and limbless segment (C). (**D** to **F**) Scanning electron microscopy image of *abd-A* crispant in first instar larvae. Prolegs are lost in *abd-A* mosaic crispants (E), and the segment shows features of the limbless segment [(F) compared with (C)]. (**G**) Expression of Dll and Abd-A proteins in wild-type embryo. (**H** to **J**) Expression of Dll and Abd-A proteins in *abd-A* crispant embryos. *Abd-A* crispants show three different types of Dll expression pattern due to mosaicism. (H) Abd-A activity was lost across the segment. (I) Abd-A activity was lost in the medial regions of the segment. (J) Abd-A activity was lost in the lateral regions of the segment. Schematic diagram shows one side of the segment (the midline of the embryo is up). Expression domain of Abd-A is colored in orange. Scale bars, 100 μm in low-magnification images and 10 μm in high-magnification images.

To test the function of the three Hox genes in limb development, we used CRISPR-Cas9. Prolegs were not affected in *Antp* crispants, but thoracic legs were, in both conserved and distinct ways relative to similar experiments in *B. mori* (see fig. S4 and Supplementary Text). *Ubx* crispant larvae displayed ectopic protuberances on the A1 and A2 segments that differed in morphology (fig. S4). Those on A1 resembled thoracic legs because of the claw at the tip (fig. S4, H and I), whereas those on A2 resembled prolegs with their row of crochets at the tip (fig. S4, J and K). Prolegs were not visibly affected. *Abd-A* crispants showed a similar phenotype as that observed in *Bombyx* RNA interference (RNAi) embryos (*32*, *33*). In mild cases, partial prolegs were retained, but in severe cases, *abd-A* crispants lost prolegs (Fig. 3, E and F). These results indicate that *Ubx* transforms thoracic legs into pleuropodia in A1 and represses prolegs in A2 and that *abd-A* is necessary for proleg development in *B. anynana*, as in *B. mori*.

To test the dual GRN hypothesis, we screened for *abd-A* mosaic mutants in embryos that only affected part of an abdominal segment. The effects of these disruptions must be examined in embryos as *abd-A* knockouts are expected to lead to ectopic pleuropodia (*26*), which will not protrude from the larval body wall, and can only be seen in embryos. To examine the possibility that prolegs are distinct traits from thoracic legs/pleuropodia, we observed embryos using Dll immunostainings that mark these two traits in different ways—pleuropodia cells are fewer and larger than those of prolegs (Fig. 3, H to J).

We observed a diversity of Dll protein patterns in *abd-A* mosaic crispants. In embryos where all the cells were lacking Abd-A in the same segment, the Dll protein acquired a pleuropodium glandular pattern throughout the abdomen (Fig. 3H and fig. S5). In segments where only the more medial region was lacking Abd-A, the Dll protein expression in a proleg pattern became patchy or disappeared together, but pleuropodia were not visible (Fig. 3I and fig. S6). In segments where only the more lateral region was lacking Abd-A proteins, the Dll expression was observed both in clusters of pleuropodium-style glandular cells and in clusters that resembled proleg epithelium primordia (Fig. 3J and fig. S7). These results indicate that prolegs and pleuropodia can develop side by side on the same segment, in a partial Hox gene knockout, and are not sharing the same cellular primordia. *Abd-A* is necessary for *Dll* expression in the proleg GRN and is also necessary for repressing *Dll* in the leg/pleuropodia GRN in the A2 to A8 abdominal segments.

To further confirm this finding, we focused on the function of *Dll* during proleg development. If prolegs and legs are serial homologs, then *Dll* should be necessary for proleg development as it is in legs (*37*). However, no *Dll* crispant embryo showed major disruptions in prolegs, suggesting that *Dll* is not involved in a major way in proleg development in *Bicyclus* as also found in sawfly prolegs (*38*).

Members of *Sp* genes, particularly *Sp5* (*buttonhead* in *Drosophila*) and *Sp6-9* (*Sp1* in *Drosophila*), are expressed in both distal and proximal parts of legs in insects (*22*, *39*) and promote appendage outgrowth through the activation of *Dll* (*22*). Furthermore, *Sp5* is expressed in abdominal appendages of *Tribolium* embryos (*23*). To test for expression of *Sp* genes in *B. anynana* legs and prolegs, we isolated all three members of the *Sp* family, *Sp1-4*, *Sp5*, and *Sp6-9* from the genome and transcriptome of this species (fig. S8). The RNA sequencing (RNA-seq) variance-stabilizing transformation counts from fifth instar transcriptomes showed that *Sp1-4* was expressed homogeneously across all tissues as also observed in other insects (*23*), whereas *Sp5* and *Sp6-9* were significantly overexpressed in legs, mouthparts, and antennae compared to prolegs, horns, and wings (fig. S9).

**Fig. 4.**
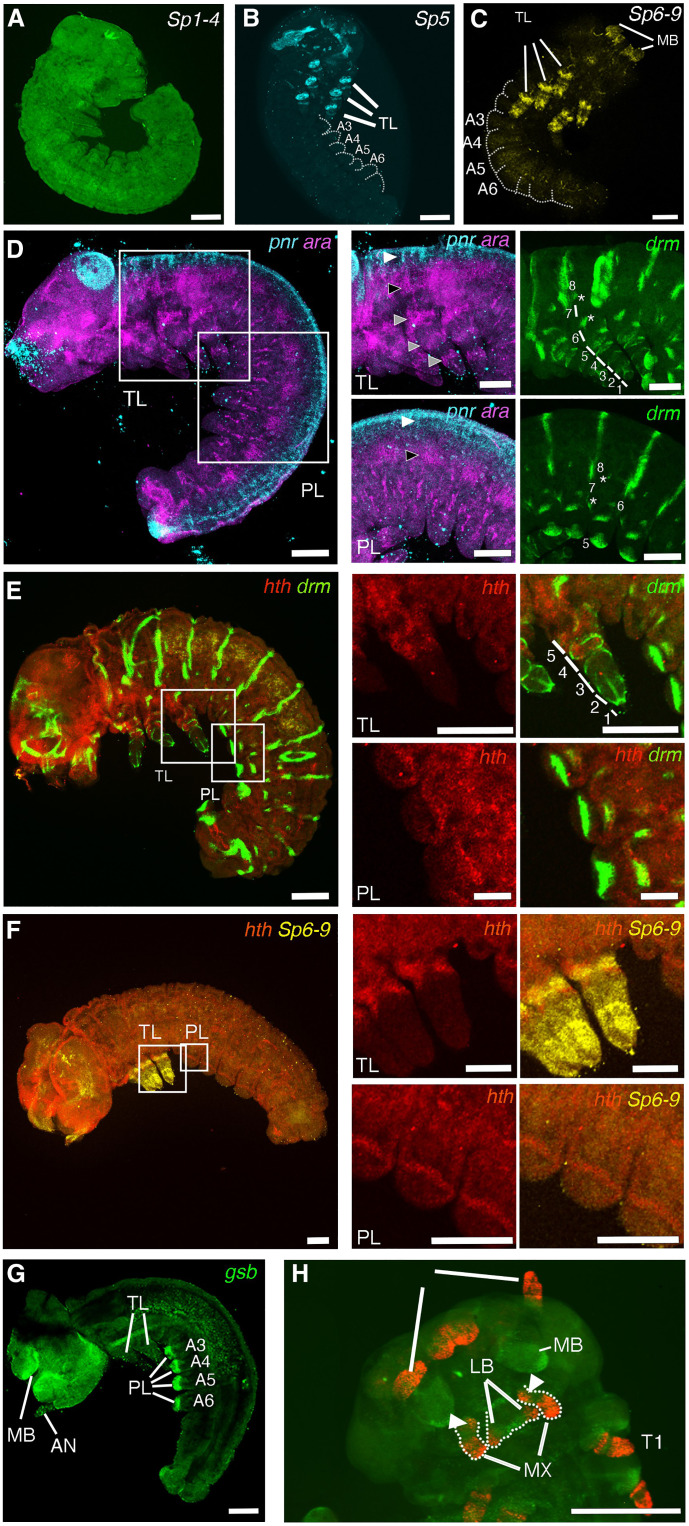
Expression pattern of leg, protopod, and endite marker genes in wild-type embryos. (**A**) *Sp1–4* is ubiquitously expressed in butterfly embryos. (**B**) *Sp5* is expressed in the thoracic legs with two armband patterns but is not expressed in prolegs. (**C**) *Sp6-9* is expressed in the thoracic legs with two armband patterns but is not expressed in prolegs. (**D**) Expression pattern of *pnr* and *ara* in butterfly embryos. In the thorax, *pnr* is expressed in the most dorsal regions of the embryo (white arrowheads), and *ara* is expressed just ventral to the *pnr* expression domain (black arrowheads) and in three bands in thoracic legs (gray arrowheads). In the abdomen, *ara* is expressed ventrally to *pnr*, but no band pattern was observed in prolegs. *Drm* is expressed in every body segment, in six armbands and two smudges in thoracic legs, and in two armbands and two smudge expression domains in prolegs. (**E**) Expression of *hth* and *drm* in butterfly embryos. *Hth* is expressed in the proximal regions of thoracic legs and in a thin stripe in prolegs. (**F**) Expression pattern of *hth* and *Sp6-9* in butterfly embryos. The proximal domain of *Sp6–9* is colocalized with *hth* in thoracic legs, but no *Sp6-9* expression was observed in prolegs. (**G**) *gsb* is expressed in prolegs and in the edge of the mandible. (**H**) Dll protein is expressed in the maxilla with two domains. White arrowheads indicate the expression domain of Dll protein in proximal regions of maxillae. Photo credit: Xiaoling Tong (Southwest University, Chongqing, China). Scale bars, 100 μm in low-magnification images and 50 μm in high-magnification images. PL, proleg; TL, thoracic leg; MB, mandible; AN, antennae; LB, labium; Mx, maxilla.

Among the *Sp* genes, *Sp6-9* is the most important in promoting leg development in arthropods (*19*, *22*, *39*–*42*), but we examined the detailed mRNA expression domains of all three *Sp* genes in *B. anynana* embryos. *Sp1-4* was ubiquitously expressed in embryos and larvae as observed in other insects [Fig. 4A and (*23*)]. Both *Sp5* and *Sp6-9* were expressed in mouthparts and in thoracic legs, with Sp5 restricted to a more proximal leg domain, but neither gene was expressed in prolegs (Fig. 4, B and C). These data suggest that *Sp* genes do not drive *Dll* expression in prolegs, as they do in legs, and *Dll* expression in prolegs belongs to a distinct GRN. These results also suggest that prolegs are not homologous to the protopod or telopod of legs, as *Sp5*/*Sp6-9* genes are markers for both.

To further confirm that prolegs are not homologous to the protopod, as proposed by Snodgrass (*5*), we examined, *ara*, a marker gene for protopod segments, including those now incorporated into the body wall in *Tribolium* and a crustacean (*19*); *pannier* (*pnr*), a body wall marker gene (*19*); *drumstick* (*drm*), a leg joint marker (*43*, *44*); and *hth*, a protopod marker in embryos (*45*, *46*). *drm* was expressed in all body segments and in six armbands and two smudge patterns in thoracic legs (Fig. 4E). Three armbands and two smudge domains of *drm* expression were observed in prolegs (Fig. 4, D and E), indicating that prolegs likely have several segments. To examine whether proximal leg segments incorporated into body wall were present in the thorax and abdomen, we examined the expression pattern of *pnr* and *ara*. In the thorax, *pnr* was expressed in the dorsal-most region of the embryo, and *ara* was expressed just ventral to *pnr* (Fig. 4D). This expression of *ara* likely corresponds to the most proximal leg segment (now part of body wall) as previously observed in *Tribolium* embryos (*19*). *ara* was expressed in three armbands in thoracic legs, with the strongest band being the subcoxa (a segment more proximal to the coxa; Fig. 4D). In the abdomen, *pnr* and *ara* are expressed in the most dorsal region, as observed in the thorax, but no banding pattern was observed in prolegs (Fig. 4D). Last, we examined the expression pattern of *hth*, another protopod marker gene. *hth* was expressed in the proximal segments of thoracic legs (Fig. 4E), while faint *hth* expression was observed in prolegs, overlapping with the proximal band of *drm* expression (Fig. 4E). To examine whether the expression of *hth* in prolegs indicates that prolegs are composed of a protopod, as proposed by Snodgrass (*5*), we examined the coexpression of *hth* with another protopod marker gene, *Sp6-9*. In thoracic legs, *Sp6-9* shows two expression domains, one in the telopod and the other in a more proximal region (Fig. 4C), with *hth* being colocalized in the more proximal domain. However, *Sp6-9* was not expressed in prolegs, indicating that the domain of *hth* expression there might be novel and part of a distinct GRN from the thoracic leg GRN. Furthermore, *ara*, normally expressed in the coxa of thoracic legs, was not expressed in prolegs (Fig. 4D). These results, again, do not support homology of prolegs to the protopod of legs, as proposed by Snodgrass.

Last, we examined the hypothesis that prolegs are homologous to leg endites (*7*). In *Tribolium*, *prd* is a marker gene for endites of mouthparts (*4*). In lepidopteran genomes, however, *prd* is not present and appears to have been replaced by a paralog, *gooseberry* (*gsb*) [fig. S10 and (*47*)]. We examined *gsb*, a paralog of *prd*, and *Dll*, both marker genes for endites of *Tribolium* mouthparts (Fig. 1A and fig. S10) (*4*). *gsb* was localized to the tips of prolegs and to the edge of the mandible of *B. anynana* embryos (Fig. 4G), as also observed in *Tribolium* mandibles (*4*). *Dll* and *hth* were observed not only in prolegs (Fig. 4F and fig. S3D) but also in endites of the maxilla [Fig. 4H and (*3*)]. This suggests that prolegs are homologous to limb endites, as proposed by Bitsch (*7*), but these endites are not emerging from the free protopod as they do in mouthparts; they appear to be emerging from the part of the protopod that is now part of body wall in insects.

## DISCUSSION

**Fig. 5.**
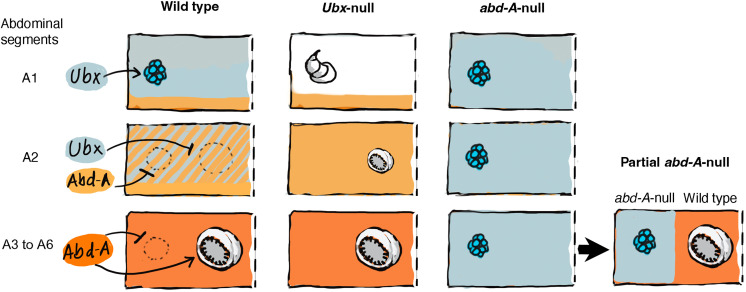
Summary of how null mutations in two Hox genes alter the development of pleuropodia and prolegs in the abdomen of *B. anynana*. Each rectangle represents half a body segment.

In this study, we have shown that two traits with different primordia and *X*-*Y* coordinates can develop in the same abdominal segment of a lepidopteran and are affected in opposite ways by *Ubx* and *abd-A*. *Ubx* is expressed in A1, where it modifies the limb GRN into a pleuropodium (Fig. 5). *abd-A* is expressed together with *Ubx* in A2, where *Ubx* represses the development of the proleg GRN and *abd-A* represses the leg/pleuropodium GRN (Fig. 5). *abd-A* is expressed alone in A3-A6, where it promotes the proleg GRN and represses the leg/pleuropodium GRN (Fig. 5). This demonstration was possible because CRISPR-Cas9 allowed the knockout of *abd-A* in just a part of each abdominal segment. In some mosaic individuals, *abd-A* knockouts removed the repressive effect it exerted on the thoracic leg primordia while keeping the gene intact in cells of the proleg primordia, where it could continue to activate proleg development. This led to the development of pleuropodia and prolegs in the same segment, suggesting that prolegs are not homologous to thoracic legs.

In addition to revealing the presence of repressed pleuropodia next to prolegs, we used RNA-seq and candidate genes to try and investigate the proleg GRN. We showed that the proleg transcriptome is most similar to head horns, rather than to thoracic legs. Prolegs also do not express important limb patterning genes, such as *Sp genes*, important for the development of whole legs (*Sp6-9*). Furthermore, no clear expression of *ara*, a coxal marker, was detected in prolegs. These results suggest that prolegs have a separate GRN from thoracic legs.

Prolegs do express the telopod marker gene *Dll*, but *Dll*’s expression at the tips of prolegs is not driven by the same cis regulatory element (CRE) that drives *Dll* in the telopod of thoracic legs. A previous reporter construct with a *B. anynana Dll* CRE (*Dll319*) drove enhanced green fluorescent protein expression in antennae, mouthparts, and thoracic legs, but not in prolegs, and when the CRE was disrupted, all traits were similarly disrupted apart from prolegs (*24*). *Dll* expression in the tips of prolegs is driven by a separate CRE, perhaps part of an endite-specific GRN. Overall, neither the RNA-seq nor the candidate gene expression data suggest that legs and prolegs are serial homologs.

Instead, the expression of genes typically associated with endite development suggests that prolegs might be highly derived endites. Prolegs expressed *gsb*, a segment-polarity gene, which belongs to the same gene family as *paired* (a pair-rule gene), which is an endite marker in *Tribolium* (*4*). *gsb* might be an interesting gene to examine in the future, in connection to endites. *Dll’s* expression in prolegs may also be connected to this gene’s functioning in endite development, as *Dll* is an endite marker in *Tribolium* mouthparts and *Artemia* thoracic legs (*4*, *20*). In addition, previous work in *B. anynana* embryos showed that prolegs and two head appendages with endites, mandibles, and maxillae express *Wnt10* at 28 hours after egg laying and *Wnt7* at 48 hours after egg laying (*48*). Because legs do not express these two *Wnts*, they might also be part of an endite-specific GRN.

We propose that prolegs are novel traits that ultimately derive from the co-option of a modular leg endite GRN to a novel location in the body. This GRN, which is still functional in the mouthparts of lepidoptera, was activated next to the repressed thoracic legs in the abdomen. Leg endites were novel traits when they first emerged in the legs of arthropods from lobopod ancestors (Fig. 1A) and therefore likely have their own separate GRN. In the ancestors of lepidoptera that did not have prolegs, the thoracic legs, to which these endites were attached, were modified into pleuropodia in A1 by *Ubx* and repressed together by *abd-A* in more posterior segments. Later, in lepidopterans, the endite GRN was reactivated by *abd-A* to produce prolegs in the abdomen. The current use of the endite GRN in mouthparts kept the GRN free of mutations throughout the evolution, allowing for its reuse both in prolegs and perhaps also in head horns.

Our data, together with that from *Tribolium*, also suggest that the repression of abdominal limbs by *abd-A* happened in a gradual way within insects. In *Tribolium*, small abdominal nubs, expressing protopod markers such as *hth* and *Sp5*, are visible in early embryos before being absorbed into the body wall (*23*, *46*). These nubs do not appear to be homologous to prolegs, as prolegs do not express *Sp5*. It is possible that abdominal limbs in *Tribolium* have been truncated at a place in the network that prevents *Dll* expression in the telopod, allowing the protopod to still differentiate into nub-like structures. In the more derived *Drosophila* lineage, the repression of abdominal limbs by *abd-A* happens at the level of an earlier enhancer (*Dll304*) that drives *Dll* expression in a small group of cells that differentiate both the coxopod and telopod of legs and wings (*30*). Given that butterflies are closer to flies than to beetles, it is possible that both lineages truncate their limbs in the same way, higher up in the limb GRN, and differently from *Tribolium*.

Our result that shows the opposite regulation of prolegs and legs/pleuropodia by *Ubx* and *abd-A*, preventing these two traits from coexisting in the same segment, also calls for a reexamination of the role of Hox genes in the development of other novelties in the body of insects (*36*). These include gin traps in the abdomen of *Tenebrio* beetles (*49*), gills in the abdomen of mayfly nymphs (*50*), horns in the thorax of dung beetles (*51*), and helmets in the heads of treehoppers (*52*), all previously proposed to be wing serial homologs. Hox genes present in these body regions could be promoting the development of these traits while also simultaneously repressing the development of wings. RNAi manipulations make it difficult to address whether both traits can develop in the same segment, as shown here for prolegs and pleuropodia, because these injections work in a systemic way, penetrating most cells in the body. The down-regulation of a Hox gene might derepress wings while perhaps failing to promote the other (potentially novel) trait. This manipulation will appear as if one trait is being transformed into the other when that may not be the case. Partial CRISPR knockouts, however, might be able to uncover whether these traits have separate embryonic coordinates, are under distinct regulation by the same Hox gene, and can develop simultaneously in the same body segment (*36*).

In conclusion, our data support the hypothesis that lepidopteran prolegs are likely novel traits, as proposed by Hinton (*9*) and others (*14*–*16*), but their developmental origin is likely derived from an old endite GRN, as proposed by Bitsch (*7*). This endite GRN has been activated in a novel way by an abdominal Hox gene in a more medial position in the embryo, next to where the same Hox gene is still repressing thoracic leg homologs.

## MATERIALS AND METHODS

### Butterfly husbandry

*B. anynana*, originally collected in Malawi, have been reared in the laboratory since 1988. The caterpillars were fed on young corn plans, and adults were found on mashed banana. *B. anynana* were reared at 27°C and 60% humidity in a 12-hour light/12-hour dark cycle.

### Short guide RNA design

Short guide RNA (sgRNA) target sequences were selected on the basis of their guanine-cytosine content (around 60%) and the number of mismatch sequences relative to other sequences in the genome (>3 sites). In addition, we selected target sequences that started with a guanidine for subsequent in vitro transcription by T7 RNA polymerase.

### sgRNA production

The template for in vitro transcription of sgRNA was made with a PCR method described in (*53*). The forward primer contains a T7 RNA polymerase binding site and a sgRNA target site (GAAATTAATACGACTCACTATAGNN_19_GTTT TAGAGCTAGAAATAGC). The reverse primer contains the remainder of sgRNA sequence (AAAAGCACCGACTCGGTGCCACT TTTTCAAGTTGATAACGGACTAGCCTTATTTTAACTTGCTATTTCTAGCTCTAAAAC). PCR was performed with Q5 High-Fidelity DNA Polymerase (NEB) in 100-μl reaction volumes. After checking with gel electrophoresis, the PCR product was purified with the Gene JET PCR Purification Kit (Thermo Fisher Scientific). In vitro transcription was performed with T7 RNA polymerase (NEB) using 500 ng of purified PCR product as a template during an overnight reaction. After deoxyribonuclease I treatment to remove the template DNA, the RNA was precipitated with ethanol. The RNA was then suspended in ribonuclease (RNase)–free water and stored at −80°C.

### Cas9 mRNA production

pT3TS-nCas9n, a gift from W. Chen (Addgene, plasmid # 46757), was linearized with Xba I (NEB) and purified by phenol/chloroform purification and ethanol precipitation. In vitro transcription of mRNA was performed using the mMESSAGEmMACHINE T3 Kit (Ambion). One microgram of linearized plasmid was used as a template, and a polyadenylate tail was added to the synthesized mRNA by using the Poly(A) Tailing Kit (Thermo Fisher Scientific). The A-tailed RNA was purified by lithium chloride precipitation and then dissolved to RNase-free water and stored at −80°C.

### Microinjection

Eggs were laid on corn leaves for 30 min. Within 2 to 3 hours after egg laying, sgRNA and Cas9 mRNA were coinjected into embryos. At that stage, the embryo is a syncytium and cell membranes will only appear around 4 to 5 hours after egg laying (*48*). Cas9 mRNA (500 μg/μl final concentration) and sgRNA (500 μg/μl final concentration) were injected. Food dye was added to the injection solution for better visualization. The injections were performed while the eggs were submerged in phosphate-buffered saline (PBS). The injected eggs were incubated at 27°C in PBS, transferred onto moist cotton the next day, and further incubated at 27°C. The hatched caterpillars were moved to corn leaves and reared at 27°C with a 12-hour light/12-hour dark cycle and 60% relative humidity.

### Immunohistochemistry for embryos

Forty-eight–hour embryos were dissected in PBS buffer under the microscope. The samples were fixed in 4% formaldehyde/fix buffer [0.1 M PIPES (pH 6.9), 1 mM EGTA (pH 6.9), 1.0% Triton X-100, and 2 mM MgSO_4_] for 30 min on ice. The samples were washed with 0.02% PBSTx (PBS + Triton X-100) three times, every 10 min, and then dehydrated with a stepwise methanol/0.02% PBSTx series from 25 to 50 to 75 to 100%. The samples were kept in −20°C. For immunostaining, the samples were rehydrated with a stepwise methanol/0.02% PBSTx series from 100 to 75 to 50 to 25 to 0.02% PBSTx, and then the samples were kept in 5% bovine serum albumin (BSA)/PBSTx for 1 hour at room temperature as a blocking reaction. The samples were replaced into the 5% BSA/PBSTx with primary antibody and incubated at 4°C for overnight. We used a rabbit polyclonal anti-Dll (at 1:200; a gift from G. Boekhoff-Falk, University of Wisconsin, Madison, WI), a mouse monoclonal anti-Antp 4C3 (at 1:200; Developmental Studies Hybridoma Bank), a rabbit anti–*Junonia coenia* Ubx antibody (at 1:500; a gift from L. Shashidhara), a mouse monoclonal anti-Ubx/abd-A FP6.87 (at 1:5; Developmental Studies Hybridoma Bank), and a rat anti–Abd-A (at 1:300; a gift from S. Tomita). The samples were washed with PBSTx three times for every 10 min. Then, the PBSTx was replaced with 5% BSA/PBSTx as a blocking reaction for 1 hour at room temperature, then replaced with 5% BSA/PBSTx with an appropriate secondary antibody (1:200), and incubated at 4°C for 2 hours. The wings were washed with PBSTx three times for every 10 min, and the wings were mounted in ProLong Gold mounting media. The images were taken under an Olympus FV3000 microscope.

### Fluorescence-based in-situ hybridization chain reaction (HCR3.0)

In-situ hybridization chain reaction (HCR) was performed on the basis of the protocol developed (*54*) with few modifications. Briefly, 48-hour embryos were placed in PBS and a small hole was made in egg cases. Eggs were fixed in 4% formaldehyde in PBS + Tween 20 (PBSTw) for 30 to 45 min and washed three times with ice-cold PBSTw for every 5 min. Embryos were permeabilized using a detergent solution (*55*) for 30 min at room temperature and washed twice with PBSTw for 5 min. Embryos were later transferred to 30% probe hybridization buffer and incubated at 37°C for 30 min. Afterward, embryos were incubated with 30% probe hybridization buffer with HCR probes at 37°C overnight. Next day, the embryos were washed four times with 30% HCR wash buffer for every 15 min at 37°C and later washed with 5X Sodium chloride-sodium citrate + 0.1% Tween 20 (5X SSCT) twice for every 5 min at room temperature. Embryos were incubated with amplification buffer and secondary probes and kept in the dark for overnight. The following day, the embryos were washed four times with 5X SSCT for every 30 min. Later, the embryos were mounted in in-house mounting media and imaged under Olympus FV3000 microscope.

### Sample collection and library preparation for RNA sequencing

To examine the transcription profiles of prolegs and other tissues, we extracted RNA from T1 legs, antennae, maxillae (mouthparts), abdomen (proleg control tissue), prolegs, horns, forewings, and hindwings from late fifth instar larvae as described in (*24*). We performed the experiment with four biological replicates per group with 10 to 20 individuals in each replicate. RNA was extracted using the QIAGEN RNA Plus Mini Kit. Twenty-five million reads per samples were sequenced with an average insert size of 250 to 350 base pairs (bp) using NovoSeq 6000 and 150-bp read length. Library preparation and sequencing were performed at GENEWIZ, China.

### RNA-seq transcriptome and annotation

RNA-seq analysis was performed as described in (*24*). The reads were trimmed and filtered for quality using bbmap tools (*56*). Processed reads were mapped to *B. anynana* genome (BaGv2) using HISAT2. StringTie (*57*, *58*) was used to produce the combined transcriptome with all libraries using the input from HISAT2 output. The final transcriptome assembled resulted in 22,689 genes with 39,534 transcripts. The transcriptome was annotated using EnTAP pipeline (*59*).

### Differential expression analysis and hierarchical clustering

Differential expression analysis was carried out using the genes read count obtained from StringTie. To verify if prolegs showed a similar expression profile to legs and its serial homologs, pairwise comparisons were performed between all the tissues, except proleg control tissue. DE (logFC ≥2 and *P* adj. ≤ 0.001) genes obtained from the pairwise comparison were used to perform HC using the run_DE_analysis.pl script from Trinity pipeline (*60*). To identify the transcription factors and DNA binding proteins, GO obtained from EnTAP annotation was used. Proleg-specific DE genes (2503) (logFC ≥1 and *P* adj. ≤ 0.05) were obtained by differential expression analysis between proleg and abdomen region using DESeq2 (data S1) (*61*). To explore the relationship between prolegs and leg serial homologs using “leg-specific” genes, we produced a list of 39 genes, which are known to be expressed and involved in the development of legs using the *Drosophila* literature (data S1). We obtained the corresponding orthologs in our current *B. anynana* transcriptome using reciprocal BLAST. We rerun the pairwise differential expression analysis, and 25 of the 39 genes were DE in at least one of the pairwise comparisons, which we used to perform HC.

## Supplementary Materials

### This PDF file includes:

Supplementary Text Figs. S1 to S11 Tables S1 to S3 Legend for data S1 Data S2 References
