# Supplementary material for: Lepidopteran prolegs are novel traits, not leg homologs
Source: Sci Adv. 2023 Oct 12;9(41):eadd9389. doi: 10.1126/sciadv.add9389 (PMC10569709; doi:10.1126/sciadv.add9389)
Supplement: Supplementary file 1 — Supplementary Text Figs. S1 to S11 Tables S1 to S3 Legend for data S1 Data S2 References [file sciadv.add9389_sm.pdf]

Science Advances
AAAS

# Supplementary Materials for

## Lepidopteran prolegs are novel traits, not leg homologs

Yuji Matsuoka *et al.*

Corresponding author: Yuji Matsuoka, matsuoka@nibb.ac.jp; Antónia Monteiro, antonia.monteiro@nus.edu.sg

*Sci. Adv.* **9**, eadd9389 (2023)
DOI: 10.1126/sciadv.add9389

**The PDF file includes:**

Supplementary Text
Figs. S1 to S11
Tables S1 to S3
Legend for data S1
Data S2
References

**Other Supplementary Material for this manuscript includes the following:**

Data S1

## Supplementary Text

***Antp* is necessary for the development of proximal regions of thoracic legs**

*Antp* crispants had short thoracic legs, lacking a tibia and a femur, showing an intermediate morphology between antennae (Supp Fig. 4B) and thoracic legs (Supp Fig. 4C). They had an unbent claw at the tip, resembling thoracic legs, but they lacked their medial segments, resembling antennae (Supp Fig. 4E and 4F). These results suggest that *Antp* is required to generate tibia and femur segments in *B. anynana* thoracic legs. The role of this gene in prolegs, however, is still unclear as prolegs were not visibly affected in our crispants

Our functional examination of the role of *Antp* in the development of the larval body plan of *B. anynana* showed a slightly different function for this gene, as compared to its function in the lepidopteran *B. mori*. *Antp* is primarily expressed in the thoracic segments in most insects and provides thoracic appendages their identity (*62*, *63*). In Lepidoptera, however, *Antp* is also expressed in a ring of cells partially overlapping with the expression of Dll in the prolegs, which develop in the A3-A6 and in the A10 abdominal segments (Supp Fig. 3A; *11*). We showed that *Antp* is required for the development of the medial segments of thoracic legs, as *Antp* crispants had short thoracic legs, lacking a tibia and a femur (Supp Fig. 4F). The role of this gene in prolegs, however, is still unclear as prolegs were not visibly affected in our crispants. In *B. mori*, RNAi against *Antp* caused fusion of thoracic segments and defects in thoracic legs (*64*), but an *Antp* (*Nc*) *B. mori* mutant showed almost complete transformation of prothoracic legs into antennae and a milder but similar type of changes in mesothoracic legs (*65*). In *Drosophila*, *Antp* mutants showed partial transformation of thoracic legs into antennae, although the distal segments developed normally (*62*). In addition, *Antp*-RNAi phenotypes in milkweed bug, *Oncopeltus fasciatus*, showed similar truncations of the femur, tibia, and some tarsal segments but still developed normal distal end segments (*63*). It is possible that the requirement of *Antp* to develop both medial and distal leg segments is a *B. mori*-specific function, whereas in *B. anynana*, *Antp* is showing the more conserved function among insects, which is the development of medial leg segments only.

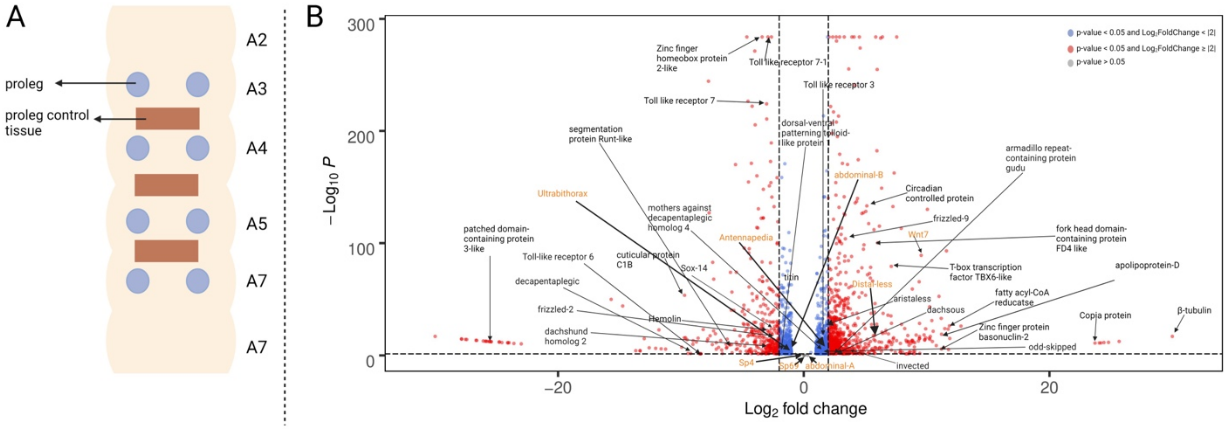


**Fig. S1. Tissue selection to identify proleg specific DE genes.** (A) Abdomen region of *B. anynana* larvae highlighting the prolegs and control tissues in between the abdomen segments used to identify proleg DE genes. (B) DE genes between proleg and control tissues, highlighting the expression level of genes studied in this work in orange color.

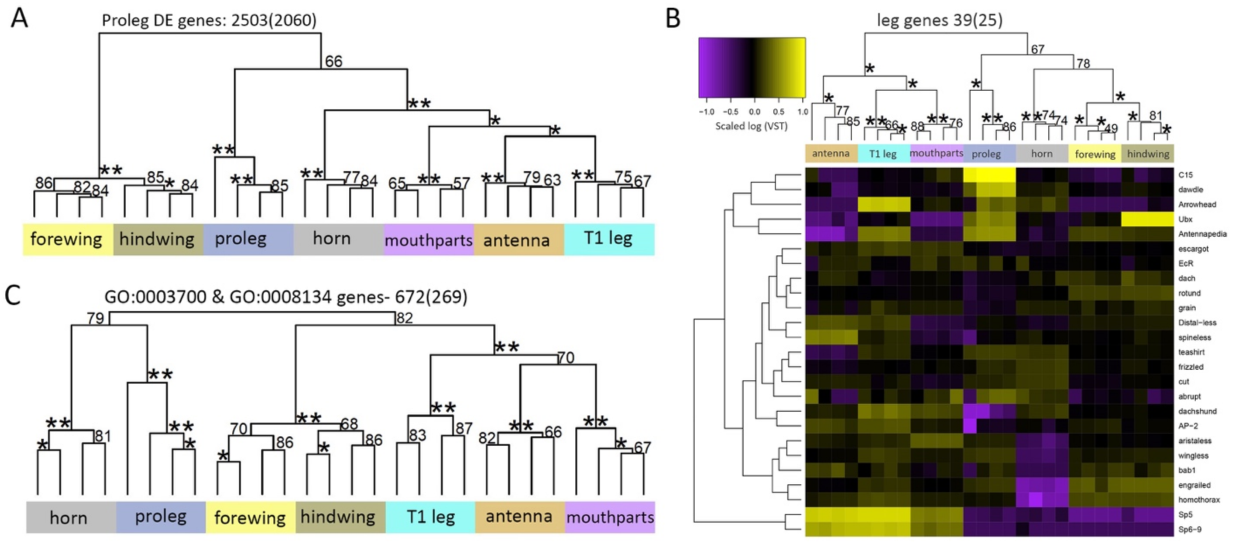


**Fig. S2. Hierarchical clustering for fifth instar larval tissue using subsets of DE genes shows proleg gene expression is different from that of legs and leg serial homologs.** (A) HC using proleg DE genes resulted in prolegs clustering as a sister clade to horns, antennae, legs, and mouthparts, with forewings and hindwings forming a sister clade to all the other tissues. (B) HC using leg genes showed prolegs clustering with horns and wings whereas legs, antennae and mouthparts clustered separately. (C) HC using DNA transcription factors (TF) and co-factors highlighted that prolegs and horns share a similar expression profile for DNA TF, clustering together and forming a sister clade to the rest of the larval tissues. The numbers mentioned in each HC heading represent the number of genes in each subset that were examined. The numbers inside the brackets represent the number of subset genes that were differentially expressed and used in the HC analyses (logFC >|2|, padj 0.001).

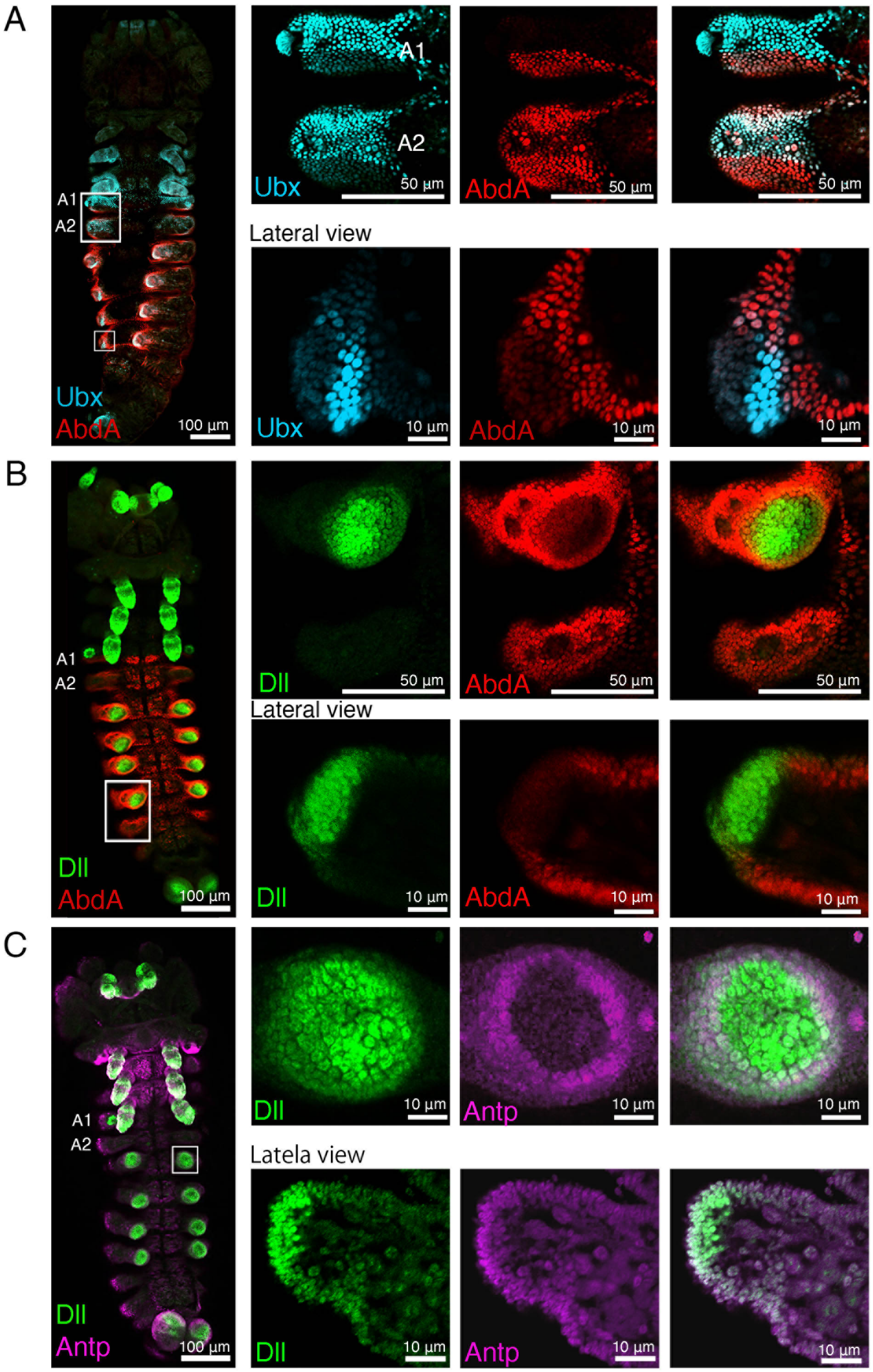

A
A1
A2
Ubx
AbdA
100 µm
50 µm
Lateral view
10 µm
B
Dll
Lateral view
C
Antp
Latela view

## Fig. S3. Expression pattern of Ubx, Antp, Abd-A, Dll in wild type embryo

(A) Double staining of Ubx and Abd-A in wild type embryos. Intense expression of Ubx is observed in T2 and T3 legs, pleuropodia, and A1 and A2 segments. Abd-A is expressed in the abdomen. In the A1 segment, Ubx and Abd-A show a clear boundary, while in A2, Abd-A is expressed across the segment, and both genes have overlapping expression domains. In prolegs, Ubx is expressed in the posterior side of the proleg tip. Abd-A is expressed at the base of the proleg. (B) Double staining of Dll and Abd-A in wild type embryo. Dll is expressed in ventral appendages, including antenna, mouthparts, thoracic legs, pleuropodia, and prolegs. Dll expression at the tip of prolegs, not overlapping with Abd-A expression. (C) Double staining of Dll and Antp in wild type embryo. Antp is expressed in the thoracic segments and prolegs. Antp is expressed at the tip of prolegs, foreshadowing the position where crochets will form. Antp and Dll expression partially overlap. White square regions were highly magnified. Scale; 100 μm.

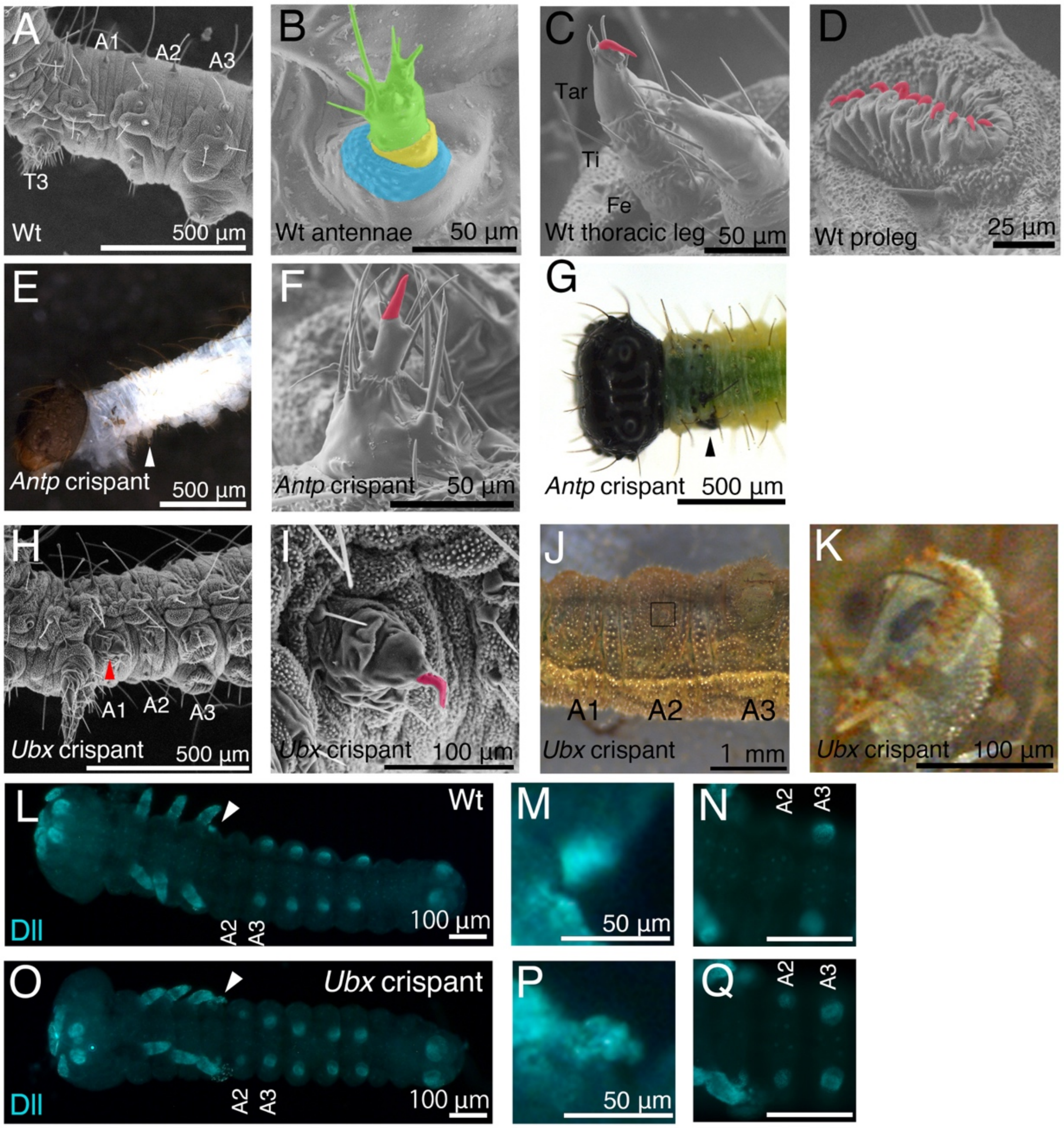


**Fig. S4. Mosaic phenotype of *Antp* and *Ubx* crispant embryos**

(A) SEM picture of a wildtype embryo. (B) SEM picture of a wildtype antenna comprised of three segments, colored in blue, yellow, and green. (C) SEM picture of a wildtype thoracic leg comprised on three segments, femur, tibia, and tarsus. The tibia has setae along its circumference and claw (red) is found at the tip of the tarsus. (D) SEM picture of a wildtype proleg. The proleg has no segments but has crochets at the tip (red). (E) SEM picture of *Antp* mosaic crispant larvae. The T2 and T3 legs of the embryo became smaller compared to the T1 leg. (F) SEM picture of thoracic leg from *Antp* mosaic crispant. The leg has only two segments, and the proximal

segment has setae along its circumference. (G) *Antp* mosaic crispant showing dark pigmentation in T2 segment. (H) SEM picture of *Ubx* mosaic crispant larvae. Ectopic protrusion in A1 segment is indicated by red arrowhead. (I) Magnified picture of the ectopic protrusion in A1 segment. The structure likely has no segments but possesses a claw at the tip. (J) Ubx mosaic crispant larva. An ectopic protrusion is found in the A2 segment. (K) Magnified region from the black square in G.    The protrusion looks like a small proleg. (L) Expression pattern of Dll in wild type embryo. Dll is expressed in ventral appendages, including pleuropodia (white arrowhead). (M) Magnified picture of Dll expression in pleuropodia. (N) Dll is not expressed in the A2 segment of wild type embryos. (O) Expression pattern of Dll in *Ubx* mosaic crispant embryo. (P) Magnified picture of A1 segment. Dll expression in the protrusion is slightly elongated compared to pleuropodia and resembles a leg. (Q) A2 segment possesses ectopic expression of Dll resembling the pattern observed in prolegs. Scale:100µm in N and Q.

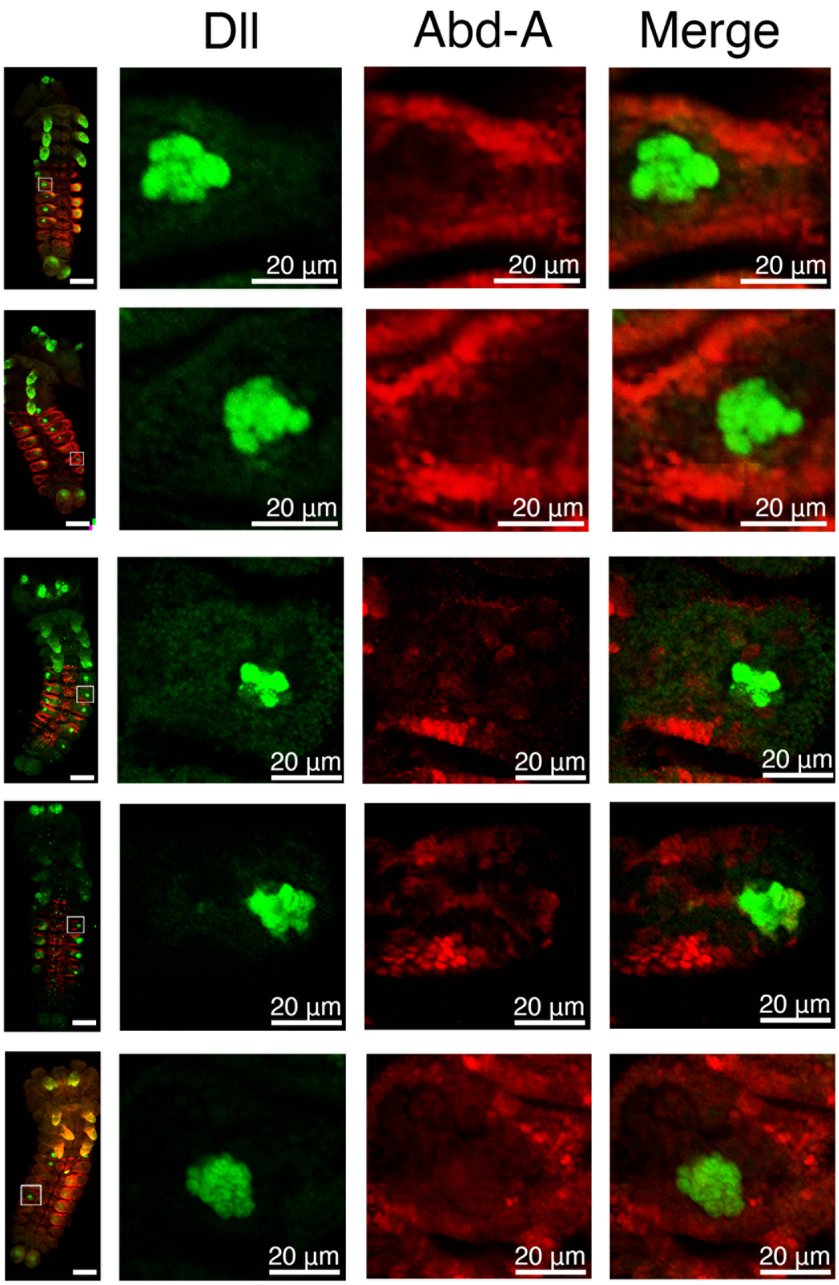

Dll
Abd-A
Merge
20 µm

**Fig. S5. *abd-A* crispant embryos showing loss of Dll expression in prolegs but acquired pleuropodia expression.**

Thirteen out of 43 *abd-A* crispant larvae loss of Dll expression in prolegs but gain of pleuropodia expression due to the loss of Abd-A activity across the whole segment. Scale:100μm in low magnification images.

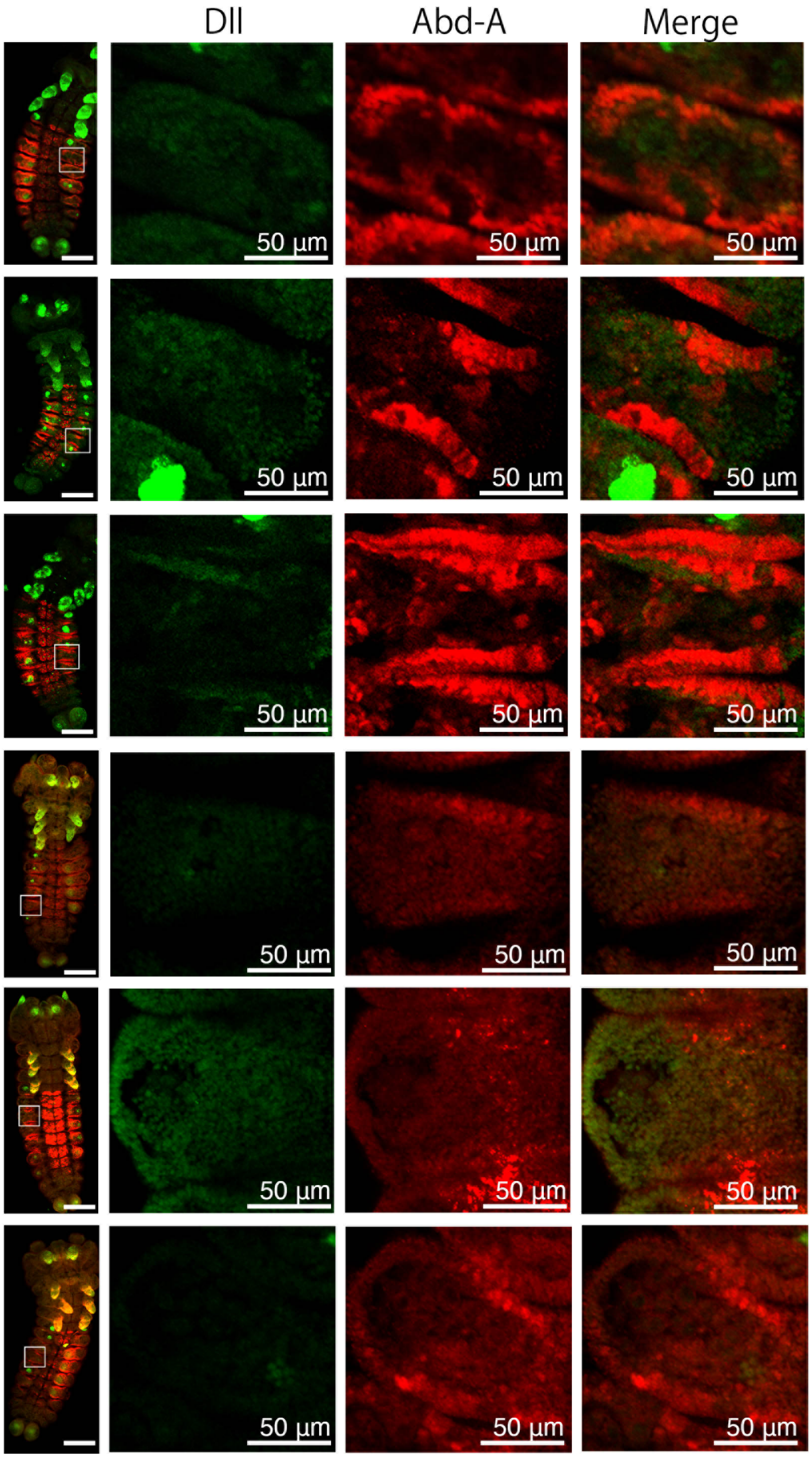
Dll
Abd-A
Merge
50 µm

**Fig. S6. *abd-A* crispant embryos showing loss of Dll expression from prolegs.**
Twenty out of 43 abd-A crispant larvae lost Dll expression from prolegs due to the loss of Abd-A activity from the mid region of the segment. Scale:100µm in low magnification images.

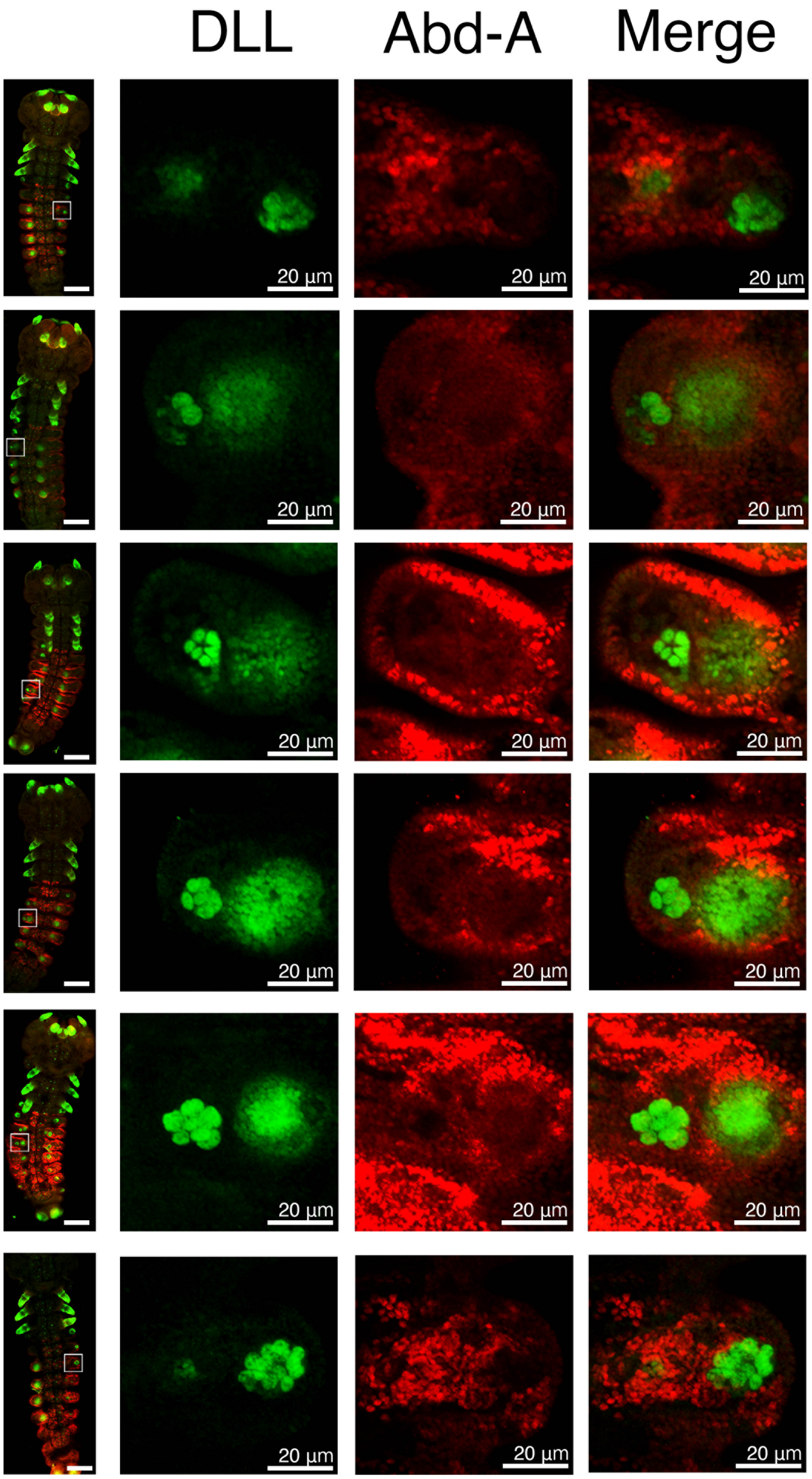
DLL
Abd-A
Merge
20 µm

**Fig. S7. *abd-A* crispant embryos having ectopic pleuropodia.**
Twenty-eight out of 43 abd-A crispant larvae acquired ectopic pleuropodia due to the loss of Abd-A activity from lateral regions of the segment.

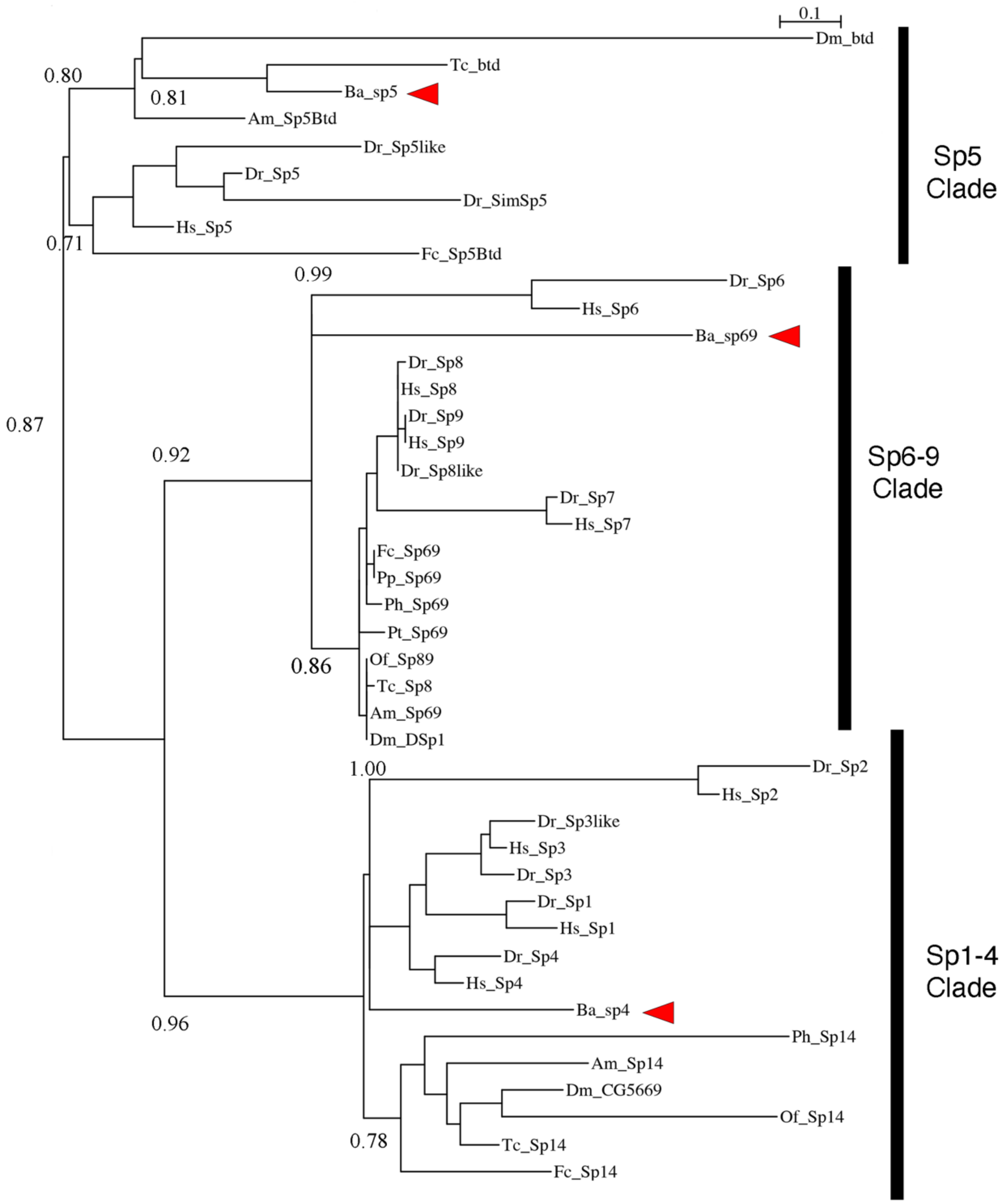


**Fig. S8. Molecular phylogenetic analysis of three *Sp* genes of *B. anynana*.**
For phylogenic analysis, protein sequence of *Sp* genes from diverse metazoan species were used following the protocol in *51*. The Amino acid sequences from the BTD-box motif to the end of third zinc finger domain were used for this phylogenic analysis conducted with PhyML in SeaView. Species abbreviations; Am: *Apis mellifera* (honey bee), Dm: *Drosophila melanogaster* (fruit fly), Dr: *Danio rerio* (zebrafish), Of: *Oncopeltus fasciatus* (milkweed bug), Fc: *Folsomia candida* (white springtail), Hs: *Homo sapiens* (human), Ph: *Parhyale hawaiensis* (Hawaiian beach-hopper, amphipod crustacean), Tc: *Tribolium castaneum* (red flour beetle). Sequence accession numbers for Sp genes are in Table S3.

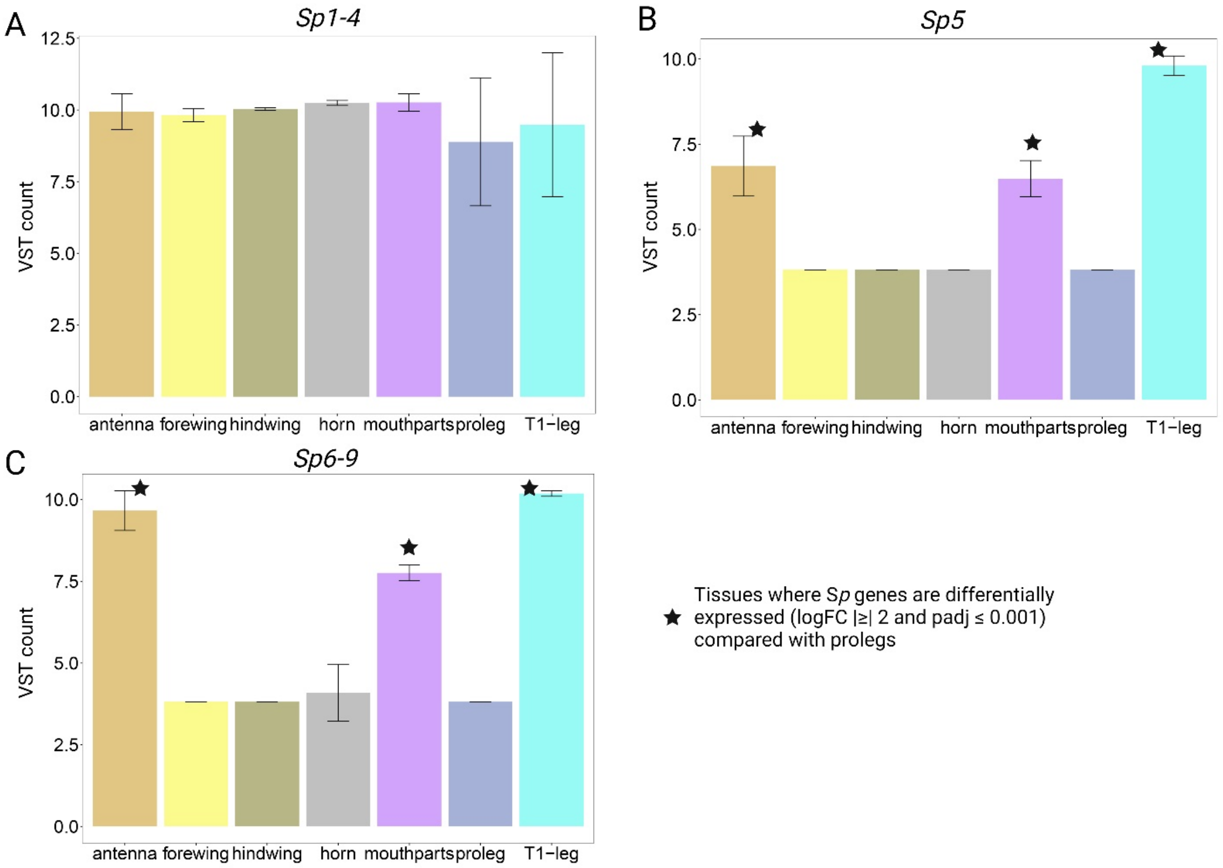


**Fig. S9. Expression level of *Sp* genes between different tissues in 5th instar larvae**. *Sp1-4* is expressed uniformly with no differences between different tissues. *Sp5* and *Sp6-9* are highly expressed in mouthparts, legs, and antennae, compared to prolegs.

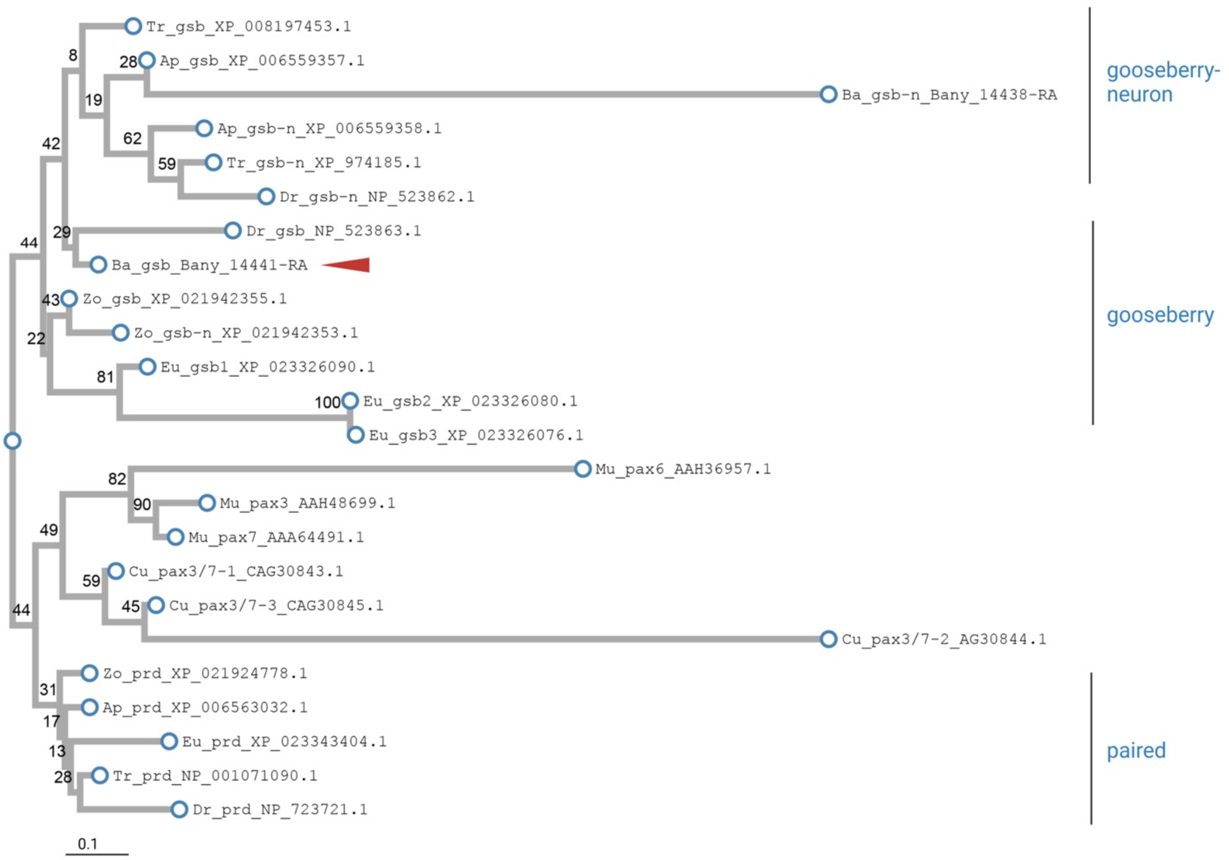


**Fig. S10. Molecular phylogenetic analysis of *gooseberry* genes of *B. anynana*.**
Amino acid sequences of paired, gooseberry, gooseberry-neuron are obtained for eight species including *Bicyclus anynana* (Ba), *Tribolium casteneum* (Tr), *Drosophila melanogaster* (Dr), *Apis mellifer* (Ap), *Eurytemora affinis* (Eu), *Mus musculus* (Mu), *Cupiennius sale* (Cu), *Zootermopsis nevadensis* (Zu). Amino acid sequences were aligned using MUSCLE in Mega7 with default parameter. A maximum likelihood tree was inferred using RAXML v8.2.11 with model PROTGAMMAJTT and default parameters implemented in GenomeNet (https://www.genome.jp/tools-bin/ete). Branch supports were computed using out of 100 bootstrapped trees.

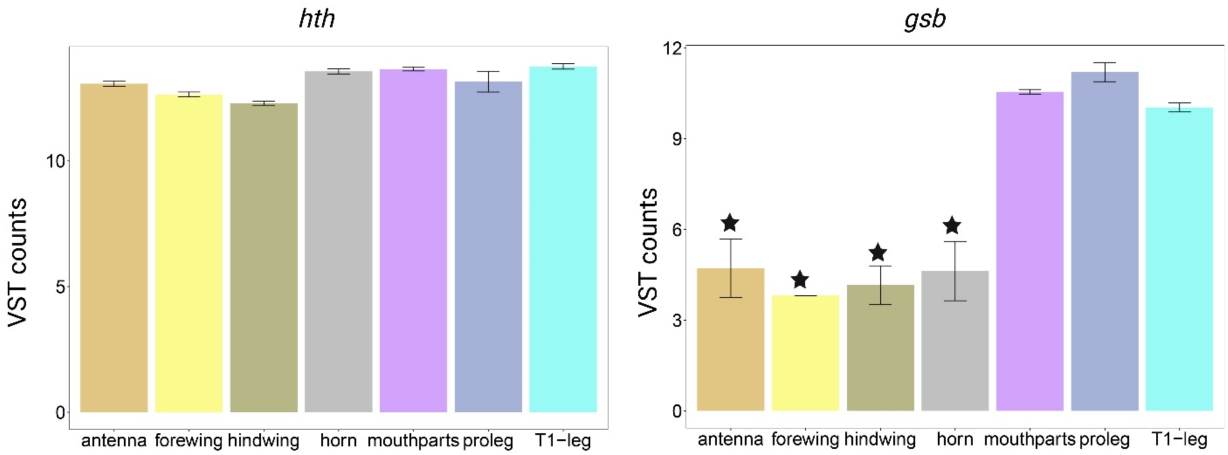


**Fig. S11. Expression level of *Hth* and *gsb* genes between different tissues in 5$^{th}$ instar larvae.** *Hth* is expressed in all tissues with no differences in expression. *paired* is expressed highly in mouthparts, prolegs and T1-legs and significantly less expressed in antennae, horns, hindwings and forewings. ( ★ - differentially expressed tissues compared with prolegs (adjp ≤ 0.001 and logFC |≥| 2).

## Table S1. Primer list

| Primer name | Sequence (5' -> 3') , (underline; PAM) |
|---|---|
| *abd-A* sgRNA | GGCGGCGCAGTTCTACCACCAGG |

**Table S2. Summary of CRISPR experiment**

| Total number of *abd-A* crispant larvae | The crispants showing both loss of proleg and ectopic pleuropodia | The crispants showing only loss of proleg | The crispants showing only ectopic pleuropodia |
|---|---|---|---|
| 43 | 13 | 20 | 28 |

Table S3. Sequence accession numbers for *Sp* genes

| | |
|---|---|
| Dm CG5669 | GenBank: NP_651232 |
| Dm Btd | GenBank: NP_511100 |
| Dm D-Sp1 | GenBank: NP_572579 |
| Am Sp1-4 | GenBank: XP_624316.2 |
| Am Sp5/Btd | GenBank: XP_001119912 |
| Am Sp6-9 | GenBank: XP_624528 |
| Tc Sp1-4 | GenBank: XP_972252 |
| Tc Btd | GenBank: NP_001107792 |
| Tc Sp8 | GenBank: NP_001034509 |
| Of Sp1-4 | GenBank: CBH30973.1 |
| Of Sp8/9 | GenBank: CAZ39568.1 |
| Fc Sp1-4 | GenBank: CBH30974.1 |
| Fc Sp5/Btd | GenBank: CBH30975.1 |
| Fc Sp6-9 | GenBank: CBH30976.1 |
| Ph Sp1-4 | GenBank: CBH30980.1 |
| Ph Sp6-9 | GenBank: CBH30981.1 |
| Hs Sp1 | GenBank: NP_612482 |
| Hs Sp2 | GenBank: NP_003101 |
| Hs Sp3 | GenBank: NP_003102 |
| Hs Sp4 | GenBank: NP_003103 |
| Hs Sp5 | GenBank: NP_001003845 |
| Hs Sp6 | GenBank: NP_954871 |
| Hs Sp7 | GenBank: NP_690599 |
| Hs Sp8 | GenBank: NP_874359 |
| Hs Sp9 | GenBank: NP_001138722 |
| Dr Sp1 | GenBank: NP_997827 |
| Dr Sp2 | GenBank: NP_001093452 |
| Dr Sp3 | GenBank: NP_001082967 |
| Dr Sp3-like | GenBank: XP_691096 |
| Dr Sp4 | GenBank: NP_956418 |
| Dr Sp5 | GenBank: NP_851304 |
| Dr Sp5-like | GenBank: NP_919352 |

| | |
|---|---|
| Dr Similar-to-Sp5 | GenBank: XP_001335730 |
| Dr Sp6 | GenBank: NP_991195 |
| Dr Sp7 | GenBank: NP_998028 |
| Dr Sp8 | GenBank: NP_998406 |
| Dr Sp8-like | GenBank: NP_991113 |
| Dr Sp9 | GenBank: NP_998125 |
| | |
| Ba Sp1-4 | GenBank: XM_024094235.1 |
| Ba Sp5 | GenBank: XM_024081517.1 |
| Ba Sp6-9 | GenBank: XM_024092868.1 |

### Data S1. (separate file)

Proleg DE genes and leg genes orthologs between *D. melanogaster* and *B. anynana*.

### Data S2. Gene sequences and HCR probes

>Hth_B1_AF546
ATGGCTCAGCCTAGGTACGACGAGAGCCTCCACGGCGGGGGCTACATGGAGGGCGGCGCCATGTACC
ACGAGCACCGGCTCACGCACCCGCACATCCCGCCGGTGCACTACCCGCCGCCCGCCGCGCCGGCGCAC
GCGTTGCCCGGCGAGCCGCTAGTGCACAAGCGCGACAAGGACGCCATATACGGGCATCCCCTGTTTCC
CCTGCTGGCGCTGATCTTCGAGAAGTGCGAGCTGGCAACGTGTACCCCCCGCGACCCCGGCGTAGCCG
GCGGTGACGTCTGTTCCTCAGAGTCCTTTAACGAGGACATCGCGGTGTTCAGTAAACAGATACGTCAA
GAAAAACCTTATTACATAGCGGACCCCGAGGTAGACTCATTAATGGTGCAAGCAATACAAGTCCTACG
GTTTCACCTATTAGAATTAGAAAAAGTGCACGAGCTGTGCGACAACTTCTGCCACCGCTACATCAGCT
GCCTGAAGGGCAAGATGCCCATCGACCTGGTGATCGACGAGCGGGAGTCAGCCCGGCCGCCGACACC
AACGGGGAGCCGCGGTCGGCGCCTGACAGCAACCACGACGGCGCATCGACCCCCGACGTCAGGCCGC
CATCGTCGTCGCTATCATACGGCGGTGCGGTGAACGATGACGTCCGCTCACCGGGCTCCGGTGGCACC
CCCGGTCCCCTCAGCCAGCCCCCGCCGCAGACCCTCGACGCGACAGATCCAGATGCCATGGGCAAATG
GTGCGGGTCGCGGCGGGAATGGTCATCCCCTCCCGACGTGGCGCGGCGGGTCTACTCCTCAGTGTTCC
TGGGCAGTCCCGGGGAATACCCAGGGGATGCCAGTAACGCGAGTATCGGCTCCGGCGAGGGTACGGG
GGAGGAAGACGACGACACGAACGGAAAGAAGAACCAAAAGAAACGGGGAATCTTTCCGAAGGTCGC
CACCAACATCCTTAGAGCGTGGCTCTTTCAGCACTTAACGCATCCCTACCCTTCGGAAGACCAGAAGA
AACAGTTGGCACAAGACACAGGGTTAACGATACTACAAGTAAATAATTGGTTCATCAACGCGAGACG
TAGGATAGTACAGCCAATGATAGACCAGTCGAATAGAGCAGTGTTCTACCCCGCAGTGTTCCCGCACG
CGGGCCCCAGCGGCGCCTACAGCCCGGAGGCCACCATGGGCTACATGATGGACGGCCAGCAGATGAT
GCACAGGCCGCCGGCCGACCCCGCCTTCCACCAGGGCTACGCGCACTACCCCGCCGAGTACTACGGAC
ACCATCTTTAA

| | |
|---|---|
| Htha_HCR_P1B1 | gAggAgggCAgCAAACggAAGCGACCCGCACCATTTGCCCATGGC |
| Htha_HCR_P2B1 | CGTCGGGAGGGGATGACCATTCCCGTAgAAgAgTCTTCCTTTACg |
| Hthb_HCR_P1B1 | gAggAgggCAgCAAACggAATTCCTCCCCCGTACCCTCGCCGGAG |
| Hthb_HCR_P2B1 | GTTCTTCTTTCCGTTCGTGTCGTCGTAgAAgAgTCTTCCTTTACg |
| Hthc_HCR_P1B1 | gAggAgggCAgCAAACggAACTGTTTCTTCTGGTCTTCCGAAGGG |
| Hthc_HCR_P2B1 | TATCGTTAACCCTGTGTCTTGTGCCTAgAAgAgTCTTCCTTTACg |
| hth3_HCR_P1B1 | gAggAgggCAgCAAACggAAACTGCGGGGTAGAACACTGCACGGT |
| hth3_HCR_P2B1 | GCGCCGCTGGGGCCCGCGTGCGGGATAgAAgAgTCTTCCTTTACg |

| | |
|---|---|
| hth4_HCR_P1B1 | gAggAgggCAgCAAACggAAGTCCATCATGTAGCCCATGGTGGCC |
| hth4_HCR_P2B1 | CGGCGGCCTGTGCATCATCTGCTGGTAgAAgAgTCTTCCTTTACg |
| hth5_HCR_P1B1 | gAggAgggCAgCAAACggAATAGTGCGCGTAGCCCTGGTGGAAGG |
| hth5_HCR_P2B1 | AGATGGTGTCCGTAGTACTCGGCGGTAgAAgAgTCTTCCTTTACg |

>Gooseberry_B3_AF488
ATTTCATATGTTCATGTTGTTACAGGACAAGGTCGTATGAATCAACTAGGCGGAGTATTCATCAACGGT
CGCCCGCTGCCCAACCACATCAGGCTGAAGATCGTGGAGATGGCGGCGGCGGGCGTGAGGCCCTGCG
TCATCTCGCGACAGCTGCGCGTCTCGCACGGCTGCGTCTCGAAGATTCTCAACAGATATCAGGTGAGA
ACTGTTTACAACCACATCAGGCTGAAGATCGTGGAGATGGCGGCGGCGGGCGTGAGGCCCTGCGTCAT
CTCGCGACAGCTGTGCGTCTCGCACGGCTGCGTCTCGAAGATTCTCAACAGATATCAGGTGAGAACTG
TTTACAACCACATCAGGCTGAAGATCGTGGAGATGGCGGCGGCGGGCGTGAGGCCCTGCGTCATCTCG
CGACAGCTGCGCGTCTCGCACGGCTGCGTCTCGAAAATTCTCAACAGATATCAGGTGAGAACTGTTTA
CAACCACATTAAGCTGAAGATCGTGGAGATGGCGGCGGCAGGCGTGAGGCCCTGCGTCATCACGCGA
CAGCTGCGCGTCTCGCACGGCTGCGTCTCGAAGATTCTCAACAGATATCAGGCGAGAACTGTTTACAA
CCACATCAGGCTGAAGATCGTGGAGATGGCGGCGGCAGGCGTGAGGCCCTGCGTCATCTCGCGACAG
CTGTGCGAAACCGGTTCAATCCGCCCCGGAGTGATCGGAGGATCAAAGCCAAGGGTGGCCACACCGG
AAGTTGAGAACCGGATAGAAGAGCTGAAGAGACAAAACCCAGGTATATTCTCCTGGGAGATTAGAGA
TAAATTAATAAAAGAAGGCATTTGTGATAAAAACACGGCCCCGTCGGTCAGTTCGATATCGCGACTCA
TAAGAGGAGGCAAAAGGGATGAATCAGACCCTAGAAGAAACCACAGTATTGATGGTATTCTAGGTCC
ATCATCATGTGAGGATTCGGATACTGAATCAGAACCAGGCATTACGTTGAAAAGGAAACAGCGAAGG
TCAAGGACAACTTTCTCTGGCGATCAGCTTGAGGCTTTGGAACGAGCATTCACTAGGACTCAATACCC
AGATGTTTATACTCGTGAGGAGTTAGCGCAGAAGACTAAATTAACTGAAGCTAGAGTTCAGGTCTGGT
TCTCTAACCGAAGAGCTCGTCTCCGCAAACAACTGAACTCTCAACAACTGAGTGCATTCAACACCATG
TCCTTACAATCTGCTTTCCCATCTGTCCATCAACAATACGAACCACCTTCTACATTTAACGCTCAATGC
GCATCATGGCAACAATCCTATTCCTCAGCTTTGGGTACCAGTTCTGTTTTAAACTCAGCATTAGCACCT
TCATTACACCAATCAGCGTTAACTGCACCATCAGTTTGCCAGTCAGCTCTTGCCGCATCATCCCTTCAT
CCTCCAACGTCTACTTCTTTCTCATCAGGAAATTTAACTCCTCTGTCCCACTCGTCTGAATTACCAACGC
CTTTGCAAGCGTCATCAGATATCACTCCTCCCAGTTCAAGCCCAACTGCGGCAAGTCCTACGGCCAATC
AGAGTGGTGGCATTACCTACCAACATCCTACTTACGCAAATGGAAGTGAAGCTCTCAGTCATCCATAT
GGATACAGCGATTACGCTAAGCAAGAAATGTCTGCCCATAACCACTGGTCGACGAGACAACTAAGTG
GGCATCCACAAAACAAACTAACGGAAGTCGGTGCGTGGCCGGAAAATTATAGTTCGTTTTTGGTACT
AACCCCTCTCACTATGCGTCACATGCGCATTCACCGAGCGAAGCTAAGTCGGGATACCCCTACATTGG
ACAATTGGGCGGCATGGACATGGGAAGAGTGCATTAG

| | |
|---|---|
| gsb_1_P1_B3 | gTCCCTgCCTCTATATCTTTTCATACGACCTTGTCCTGTAACAAC |
| gsb_2_P1_B3 | gTCCCTgCCTCTATATCTTTCGCACAGCTGTCGCGAGATGACGCA |
| gsb_3_P1_B3 | gTCCCTgCCTCTATATCTTTTCTCCCAGGAGAATATACCTGGGTT |
| gsb_4_P1_B3 | gTCCCTgCCTCTATATCTTTTGTGGTTTCTTCTAGGGTCTGATTC |
| gsb_5_P1_B3 | gTCCCTgCCTCTATATCTTTGTAAGGACATGGTGTTGAATGCACT |
| gsb_6_P1_B3 | gTCCCTgCCTCTATATCTTTGAGTTAAATTTCCTGATGAGAAAGA |
| gsb_1_P2_B3 | CGTTGATGAATACTCCGCCTAGTTGTTCCACTCAACTTTAACCCg |
| gsb_2_P2_B3 | TCACTCCGGGGCGGATTGAACCGGTTTCCACTCAACTTTAACCCg |
| gsb_3_P2_B3 | TGCCTTCTTTTATTAATTTATCTCTTTCCACTCAACTTTAACCCg |
| gsb_4_P2_B3 | ATGATGGACCTAGAATACCATCAATTTCCACTCAACTTTAACCCg |
| gsb_5_P2_B3 | GTTGATGGACAGATGGGAAAGCAGATTCCACTCAACTTTAACCCg |
| gsb_6_P2_B3 | TTGGTAATTCAGACGAGTGGGACAGTTCCACTCAACTTTAACCCg |

>Sp6-9_B2_AF647
ATGGCGAAAGCGGTTCTCATGTCATTTGTTGCTAACTTCACAAACATAATGTCACCTCAGACGCGCGA
GTCACCTCAGACGCGCCTCGCCTGTGTCGATACGTTCAATATCCGATACGCAACAAGAAATAAAATAT

CGGAACATGCCTTGCATTCTATAAAGATGCAGCGATGTGACATCGCTCATTTTCATGGCAACTACCTGG
AACACCCGAGCTTACGCGGCACGCCGCTGGCGATGCTCGCGGCGCAGTGCAACAAGCTGTCGAATTTT
TTGACAGACCAGGAAAAGGCCCATCAAAATAATCGCGACGAGGCCGGCGCCGTTTTCAGTCGGCTCG
AGCTCGAACCGCACTTTGATGCCAATCTGGTGGTTGAGTTGACGACCAATACCTATATCAAAATTCTG
AACCCCGCGCCCAAATGGAGCATAATGCCTAGGGCTTCGGCTGTGGCCTGTGCGATTTCGTGGGAAAC
CGAAAGGGTTGTGGATGTTTCAGAATACCAGATACTTGAAATCACATCCCTAGTAGCCCGCTCCCCCA
TCATTTTAACATCAGTATGTTGTCTTTCTTTACAGGAACACCCGAGCTTACGCGGCACGCCGCTGGCGA
TGCTCGCGGCGCAGTGCAACAAGCTGTCGAGCAAGTCTCCGCCGCCGCTCGCCGACGCGGCCGTCGGG
AAAGGATTCCATCCGTGGAAGAAGAGCCCCGGCACGCACTCCCCGCCCGGCGCCGGCCTCGCGCCGC
GCGCCCAGCCGCCCGCCTGCGCGGCGTACGCGCGTGCTCCCACCTCGTGCGCCGCCGCGCCCGCCTAC
GGCAACGACCTGTACTTCCCCTCGTCCGGCGACCAACTCCTCGGCAAAAGCGAATCGAGCGCGAGCCT
CGGCTCCATGTACTCGAGGCATCCCTACGAGTCGTGGCCCTTCAACGTCGGCACAGGAGGCAGCAGCG
GCGCGCTCAAAGCTGCCGAAATGGGCGGCGTCAGCGCCGTCGGCAGCGCTTGGTGGGACGTTCACAG
CGGATGGCTCGACGTAGGAGGACAGATGGGAAAAGTGTACGGAAAGACGTCCCACCTAAAAGCGCAC
TTACGCTGGCACACCGGCGAGAGACCGTTCGTGTGCAATTGGTTATTTTGTGGCAAACGGTTTACACGT
TCGGACGAGCTGCAGCGGCATTTGCGAACGCACACGGGCGAGAAAAGGTTCGCGTGTCCGGTGTGCA
ACAAGCGGTTCATGCGGTCGGATCACCTCGCGAAGCACGTGAAGACGCACAACGGAGGCAAGAAGGG
CAGCTCGGATTCATGTTCAGACTCAGAGGAGAACAGTCAAGGCGAGAGCGGTCTCGGGGGCCGGTCG
CCGGAGCACCCGCTGGACGTGAAGCCGGGCACGCTGGTGTGACGCGGGGGCGCGGCGCTCTGCGTGC
CGCACCATGCCTCGCTCCTGTGATACCCTGGCCGCGCGACCGGCCGCCGCCTGTACATAGCGACATAT
CAAACACTGTGTAACATTCTGTGTAAATTGTGAATTGAAGACTTACTAGTAGCAGTGTGAACGCTGCC
TTAATAGTGAATTGTGAGATTTGAAAGTGTACAGTTTAGAAATAGATATTATTAAATATTGTCTATTAA
TAGTAATAACTGGATATTATATTATAATTGATATAGAATAAGTAGAATCGCACTGTTAGTCCGAAATC
GCAGTTGTAATTTATTTAAATAATTACGGTTTACCTTCTTAAGGTGAATTCTTGTAATACCGTGCAGGA
GCCGGACTGGACGATTTTGTGTACTTACACTCACGACCGGAGATACCCAGACTGTACTGTACCAGAGT
AAAGATACCTACTGTACACTCAGGTCAAAACACCCTTTCCGAAAGACCCTCGCCGAAGCCGAGGTCCG
AGTCACAGAGGAGACGCCCTTAGAGAGTCTCTGCTCTGCTTCTGCCTTCGGGCCCCATCAGGCGATAC
TTTTGCGCTCGCTGCACAATAGACAAATTTCAACCAATAGTGGTGCAACGGGAAATACTCTACCTATA
TCTTTCATTGCATTGGGACGGAAGAGATTTTGACGTTTTGTCTAAAACTCTTCACTTGGTCGCATGTTA
GCTCTAATGGTCACCGTACGTAGGTATCGTGTAAACTTTTGAGAAAAGTTGTGGTTGTACTGTCATATT
TTCGTTGTCAAATAAAATTGTACTATCCATCGAAGGTTCTACACAAAAAAATTGAGTCGAGGCGTCCA
CAATTTCGCATCGTACGCATTGGACGCATCGAATCAAACGGATTTTAGTATTCATTGTATAGAAAGTCA
AACAAGTGCGTCCACTGATCCGCGTCGTACGAAATGCATCGAACGGATTTTGTCTACTGTAAAAATTA
AAAGTCAGTTGGATGCAATGCGTCCTATACGTACGATGCGGATAAGTGGACGCCTACCTTTGGAAACC
ACTGTCCTTGGCCTGCACGATCTTACCTGAATCGACCTTTATTTGTAAAGTAAGATATTATTAACATTT
CTATAATAATTACCTCAGCAAAAACAATAGCAAATTGTATTTATATTATATTTTTGTCTATACCTAATA
TAATTATTTCATTGTTAATTTTTAAATCACATTTAATAGAAGTTTATGTATGAAAATACTGTTCAAATA
AATCGTTAGCAGATACTTCAACAGTATACAAATTGATATTACCTTTAAAACGGTTCTTTTTAGGGTTCC
ATACTCCAAAAGGAAAAACGGAACCCTTCTAAGATCACTCTGTCCTAAAATAAAGGGCCTTAACCCTT
TAACTCAGGAGTACGTGGAGGTATCAAGTTGAAATTTAATCCGTATAGGTACTCAAATATACGGTCCC
ATGAAGTTGTATAAAATAAATCTTCTAAGTTTACGTAAAAAAATATATGGCCGTTTATTTAGCAAAAT
AAAGAATATCGTCGTATCGTATCGACACTCTCAAGGAATTCAAAATTTCCCGTTGACCTAGAACTATG
AAACTTGACAAGTAACATTCATTATACAAGAAAATACAAGTACTTATACAAGAAAAAATCTCAAAAC
ATACATTTGTAATTAAATCTTAAAAAACAGGTAACAGAAAATCTGTAGCGCGGGTTAGAAGTGTCTGT
ACCTAAGCTTAAGTAAAACGAATTGTTATTTTAATGGTTGCCGATGAACTATTGTAAAAAGTGACAAA
CCGATTAGAAACTAGGAACAAGAACAGTTTTTTTAATAACATATTTTATTTATAAGATAAATCTGATA
AATAAGATAAT

| | |
|---|---|
| Sp6-9_1_HCR_P1B2 | CCTCgTAAATCCTCATCAAAGTTAGCAACAAATGACATGAGAACC |
| Sp6-9_1_HCR_P2B2 | CGTCTGAGGTGACATTATGTTTGTGAAATCATCCAgTAAACCgCC |
| Sp6-9_2_HCR_P1B2 | CCTCgTAAATCCTCATCAAATGAACGTATCGACACAGGCGAGGCG |
| Sp6-9_2_HCR_P2B2 | TTTTATTTCTTGTTGCGTATCGGATAAATCATCCAgTAAACCgCC |
| Sp6-9_3_HCR_P1B2 | CCTCgTAAATCCTCATCAAATCACATCGCTGCATCTTTATAGAAT |
| Sp6-9_3_HCR_P2B2 | AGGTAGTTGCCATGAAAATGAGCGAAAATCATCCAgTAAACCgCC |
| Sp6-9_4_HCR_P1B2 | CCTCgTAAATCCTCATCAAAGCCAGCGGCGTGCCGCGTAAGCTCG |
| Sp6-9_4_HCR_P2B2 | AGCTTGTTGCACTGCGCCGCGAGCAAAATCATCCAgTAAACCgCC |
| Sp9_5_HCR_P1B2 | CCTCgTAAATCCTCATCAAACACGAAATCGCACAGGCCACAGCCG |

| | |
|---|---|
| Sp6-9_5_HCR_P2B2 | GAAACATCCACAACCCTTTCGGTTTAAATCATCCAgTAAACCgCC |
| Sp6-9_6_HCR_P1B2 | CCTCgTAAATCCTCATCAAAGCTACTAGGGATGTGATTTCAAGTA |
| Sp6-9_6_HCR_P2B2 | ACTGATGTTAAAATGATGGGGGAGCAAATCATCCAgTAAACCgCC |
| Sp6-9_7_HCR_P1B2 | CCTCgTAAATCCTCATCAAAGGTGCGGCACGCAGAGCGCCGCGCC |
| Sp6-9_7_HCR_P2B2 | GGCCAGGGTATCACAGGAGCGAGGCAAATCATCCAgTAAACCgCC |
| Sp6-9_8_HCR_P1B2 | CCTCgTAAATCCTCATCAAACAGTGTTTGATATGTCGCTATGTAC |
| Sp6-9_8_HCR_P2B2 | AATTCACAATTTACACAGAATGTTAAAATCATCCAgTAAACCgCC |
| Sp6-9_9_HCR_P1B2 | CCTCgTAAATCCTCATCAAATTCACTATTAAGGCAGCGTTCACAC |
| Sp6-9_9_HCR_P2B2 | TAAACTGTACACTTTCAAATCTCACAAATCATCCAgTAAACCgCC |
| Sp6-9_10_HCR_P1B2 | CCTCgTAAATCCTCATCAAACCAGTTATTACTATTAATAGACAAT |
| Sp6-9_10_HCR_P2B2 | TATTCTATATCAATTATAATATAATAAATCATCCAgTAAACCgCC |

>Sp5-buttonhead_ B2_AF647
ATGCAATACGTGGATCCGCCCGCGCAACAGATGATGAACGTAATGAGCGGCTACGGTTGCGGCTACG
GGCGCAGTGACGCGCTGTCACCCGCCTCCGACTTGTCCTCATGCAGCAGCGCTTCCTCGGGCGCGTGG
TGCGGCGCCCCCTGGCGGGAATTGCCGCCTTATCCACAGTACTCGCCGTACTGGCCCGCGGCGACCGC
CCCGGAGGACGCCCGCGAGTTGCAGCGCCGCTGCGCGAAGTGCCGCTGCCCGAACTGCCTCACCGAA
GCGGCAGGCTTCGGCCCCAACTTCGGCAAGGACGGTGCGAAACGAGAACACGTCTGTCATGTGCCCG
GTTGTGGAAAAGTTTACGGAAAAACGTCACACTTAAAGGCCCATCTTCGTTGGCACACCGGCGAGCGA
CCTTTTGTCTGCAACTGGCTGTTCTGCGGAAAAAGATTCACTCGCTCAGATGAGCTGCAGCGACACCTC
CGCACTCACACCGGAGAGAAGCGATTCGCTTGTCAATTGTGCACTAAACGTTTCATGCGCTCCGACCA
CCTCGCGAAACATGTGAAGACTCACGCTAACGTTACGAGGAAATCGAAGAAAGCTAAAGAAGACGAC
GCGAAAGCGGACTCCCCGAAACCGAGCGATGAAAAAGTAGAGAAGCAACCCAACGAGACAGTTGTTC
CAGCTGTGTCAATCGGAAGTTCGCATTACGGCACGGTGCCAGCGGTGACTGCGCCTAAACAGATGTTG
AACTACGGCACAGTAGCTCCGCAAGTGATGCAGGGTACTCCGGCTACTTACAACAATAATGCTGTGTT
TACAAGCGGCAGCGTTATGTACGGAAGCGGTTGGTGTCCGGAGATGCGTCAAGAGTCTTACTACGCTC
GTCCGCCAGCTAGAGACCCTCGGCTCTACCAGCAGTACACACCGCTCACAGCCTACCAGTGCGGTTCT
AAGGATAACAGTTACGCCATGTTCCAAGGACATTACAACTTACAGCCGCCGGTGGCCATTGGACAATA
A

| | |
|---|---|
| Sp51_HCR_P1B2 | CCTCgTAAATCCTCATCAAATCTGTTGCGCGGGCGGATCCACGTA |
| Sp51_HCR_P2B2 | AACCGTAGCCGCTCATTACGTTCATAAATCATCCAgTAAACCgCC |
| Sp52_HCR_P1B2 | CCTCgTAAATCCTCATCAAAACAAGTCGGAGGCGGGTGACAGCGC |
| Sp52_HCR_P2B2 | CGCCCGAGGAAGCGCTGCTGCATGAAAATCATCCAgTAAACCgCC |
| Sp53_HCR_P1B2 | CCTCgTAAATCCTCATCAAATACTGTGGATAAGGCGGCAATTCCC |
| Sp53_HCR_P2B2 | GCGGTCGCCGCGGGCCAGTACGGCGAAATCATCCAgTAAACCgCC |
| Sp54_HCR_P1B2 | CCTCgTAAATCCTCATCAAAGGCACTTCGCGCAGCGGCGCTGCAA |
| Sp54_HCR_P2B2 | CCGCTTCGGTGAGGCAGTTCGGGCAAAATCATCCAgTAAACCgCC |
| Sp55_HCR_P1B2 | CCTCgTAAATCCTCATCAAACGCGTCGTCTTCTTTAGCTTTCTTC |
| Sp55_HCR_P2B2 | ATCGCTCGGTTTCGGGGAGTCCGCTAAATCATCCAgTAAACCgCC |
| Sp56_HCR_P1B2 | CCTCgTAAATCCTCATCAAACAGCTGGAACAACTGTCTCGTTGGG |
| Sp56_HCR_P2B2 | TGCCGTAATGCGAACTTCCGATTGAAAATCATCCAgTAAACCgCC |
| Sp57_HCR_P1B2 | CCTCgTAAATCCTCATCAAATGCCGTAGTTCAACATCTGTTTAGG |
| Sp57_HCR_P2B2 | TACCCTGCATCACTTGCGGAGCTACAAATCATCCAgTAAACCgCC |
| Sp58_HCR_P1B2 | CCTCgTAAATCCTCATCAAAACGCTGCCGCTTGTAAACACAGCAT |
| Sp58_HCR_P2B2 | TCCGGACACCAACCGCTTCCGTACAAAATCATCCAgTAAACCgCC |
| Sp59_HCR_P1B2 | CCTCgTAAATCCTCATCAAAGGGTCTCTAGCTGGCGGACGAGCGT |
| Sp59_HCR_P2B2 | AGCGGTGTGTACTGCTGGTAGAGCCAAATCATCCAgTAAACCgCC |
| Sp510_HCR_P1B2 | CCTCgTAAATCCTCATCAAAGGAACATGGCGTAACTGTTATCCTT |

| | |
|---|---|
| Sp510_HCR_P2B2 | CCGGCGGCTGTAAGTTGTAATGTCCAAATCATCCAgTAAACCgCC |

>Sp1-4_ B2_AF647
ATGTCGTCTGAACCCTGTAAAGTGACAGTGGAATACATTAGTGAGGACAAAATAATAAAGGAGGGTA
GCGGCACCCAGTCGCCCCTGGCGTTGGTGATCAGCACTGCTGTTGTGCAGCAATTGCGGGCGCAAGGA
TTAACGGGCAATGATCTATCAGCAGCGATAGTAAATGCGGCCAGCACTGTACCTAATGGCATACAGGT
GCAGCAACCTCAGGTGATATCAATGCAGCAGCTGCAGTCGCTTCTGGGCGGCGGCGTGGTGTCCGGCT
CCGAGGGTGCCACGTACCAGAACGCGCCACAGCAGCTGCTCCAGATCCACCCCCAGCTCTTGCAGCAA
CAAGCAGGCAATGTGTACGGGGGTTGCATGGTGGGCGGGGTGGCGCCGCTGCAGGCTGTCACCGTGG
ACGGTCAGGAGGCGCTCTTCATACCCTCACATCACGCTCAGAATTTCTCGGGAATGGGCCAGATGAGC
TTAGTCAACGGTCAGTTAGTCAGGACGCCCGTGCTGCCGACGGGTTTCCTACAAAACGTCATGCATCT
ACCAACAGAGCAACAGGCGACCGTCACCATCCCCGGTACTAACATAACCATCCCGCTCAGCGCACTCG
CCGGGAACCAGATGATCACTATACCGGGGACCAACATATCCATCCCCGGCGGTATACAGATACCGACG
AGCCAGCCCATTACCATACCTAGCCCCGTCCAGCTGCCGGTGGCGAGTGTGGCGAACATGGCCAACAT
GGCCAACGTCACCAACGTCGCCACGGTGGCCAATGTGGCCAACGCTGGCGTCAGCTTGGCGACCGGG
GACGCCAAGAATGGGGGGAATGGAAAAGAGAGCAAATCGCCCGACAGTCCTAACTCGCAAGGCGGG
GGCGTGGCCGTGCGCGGCGGCATGGGCGGCGTGGGCGTGGTGCCCGTGCAGGTGCCCATGCAGGCCC
AGCCGCAAGTCGCCAACGTTCTGACTCCGTCCGGTCAAATACAGCAGATACAGATAGCGTCGCTTGGC
AATGTGCAGCAGGCTGGTGCCCCCCAGCAGGCCGCCGCGACGGCGGGGCCCCCCACCATCACTCTACA
GGCGGTGGCGGGCCAAATGCAACCGGACACGGCTCAAGTGCAACAGCCGCAAGCGATTATCACATCC
ACGCCTGCGGGGCAGCAGGTCACCGTTATACCGACTAGCAATGTGACGACCGCCAATGTGGTGCAGGT
ATACGTCTGCGTTTGCGATCGTAAAAAGGAAATCGACGTGCCACTTGGCTACAGGTTCAGGGCCGTGT
TAGGCTACGGCAAACGTCTGGTGAACAGGGCCTTGTTGGACTACGGCAAACGTCTGGTGAACAGCGCC
TTGTTGGACTACGGCAAACGTCTGGTGAACAGGGCCGTGTTAGCTTACGGCAAACGTCTGGTGAACAG
GGCCGTGTTAGCTTACGGCAAACTTCTGGTGAACAGCGCCTTGTTGGACTATGGCAAACGTCTGGTGA
GCAGCGCCTTGTTGGACTACGGCAAACGTCTGGTGAACAGGGCCGTGTTAGCTTACGGCAAACGTCTG
GTGCCGACGCTAGGCGTGGGCGGGCTGGGCGGCGCGGTGCAGCTGGTCCCAGCGATGGGCCTGCCGG
GGGCGGTGCAGCTCACGCAGATACCGCAGGCGCAACAGCAGGCACAGCCGCAGCCTCTTATTGGACA
GCAGATCCAGCAGGACCCCAACGAGCCGGGCAAGTGGCAAGTGGTCACCGTGAGCGCGGGCAGCACC
GCCACCACGGAGTGCGAGGCGGAGAAGCTGCGGCCCACCAGCCCCAACGGCGGCAAGCGGCTCATGA
AGCGCGTGGCGTGCACGTGTCCCAACTGTGACCAGGGGGAGAACCGGCTGGTGGACCGCAAGAAGCA
ACACCTGTGCCACATCCCGGGCTGCAACAAGGTGTACGGCAAGACGTCGCACCTCCGCGCTCACCTGC
GCTGGCACTCCGGGGAGCGGCCCTTCCTCTGCAACTGGCTGTTCTGTGGCAAGAGGTTCACGCGGTCA
GATGAGCTGCAGAGGCACCGGCGCACGCATACGGGCGAGAAACGCTTCGAGTGCCCCGAGTGCAGCA
AGCGGTTCATGCGCTCCGACCACCTCGCCAAGCACGTGCGCATACACACCAAGAACCGGATCACGGA
GGTAGCGACGTCGACGCAGTCCATGTATTCGGACTCGGGCGACGACAGCTGCGACGAGAAGATGATG
CTCACCATAGAGACCATGCAGGTGCCTGAGAATGAGGAGAAACTGGTCATGATTAGGCCGGGCCTCA
AGATGGAGCCCGACCATATCGACAGCTAG

| | |
|---|---|
| Sp1-4_1_HCR_P1B2 | CCTCgTAAATCCTCATCAAATATTCCACTGTCACTTTACAGGGTT |
| Sp1-4_1_HCR_P2B2 | TCCTTTATTATTTTGTCCTCACTAAAAATCATCCAgTAAACCgCC |
| Sp1-4_2_HCR_P1B2 | CCTCgTAAATCCTCATCAAATGATCACCAACGCCAGGGGCGACTG |
| Sp1-4_2_HCR_P2B2 | CCCGCAATTGCTGCACAACAGCAGTAAATCATCCAgTAAACCgCC |
| Sp1-4_3_HCR_P1B2 | CCTCgTAAATCCTCATCAAATATCGCTGCTGATAGATCATTGCCC |
| Sp1-4_3_HCR_P2B2 | ATTAGGTACAGTGCTGGCCGCATTTAAATCATCCAgTAAACCgCC |
| Sp1-4_4_HCR_P1B2 | CCTCgTAAATCCTCATCAAATGCATTGATATCACCTGAGGTTGCT |
| Sp1-4_4_HCR_P2B2 | CCGCCGCCCAGAAGCGACTGCAGCTAAATCATCCAgTAAACCgCC |
| Sp1-4_5_HCR_P1B2 | CCTCgTAAATCCTCATCAAAACCCCCGTACACATTGCCTGCTTGT |

| | |
|---|---|
| Sp1-4_5_HCR_P2B2 | CAGCGGCGCCACCCCGCCCACCATGAAATCATCCAgTAAACCgCC |
| Sp1-4_6_HCR_P1B2 | CCTCgTAAATCCTCATCAAAATGAAGAGCGCCTCCTGACCGTCCA |
| Sp1-4_6_HCR_P2B2 | GAGAAATTCTGAGCGTGATGTGAGGAAATCATCCAgTAAACCgCC |
| Sp1-4_7_HCR_P1B2 | CCTCgTAAATCCTCATCAAACGCCTGTTGCTCTGTTGGTAGATGC |
| Sp1-4_7_HCR_P2B2 | TATGTTAGTACCGGGGATGGTGACGAAATCATCCAgTAAACCgCC |
| Sp1-4_8_HCR_P1B2 | CCTCgTAAATCCTCATCAAAGATCATCTGGTTCCCGGCGAGTGCG |
| Sp1-4_8_HCR_P2B2 | GATGGATATGTTGGTCCCCGGTATAAAATCATCCAgTAAACCgCC |
| Sp1-4_9_HCR_P1B2 | CCTCgTAAATCCTCATCAAAGGTAATGGGCTGGCTCGTCGGTATC |
| Sp1-4_9_HCR_P2B2 | CACCGGCAGCTGGACGGGGCTAGGTAAATCATCCAgTAAACCgCC |
| Sp1-4_10_HCR_P1B2 | CCTCgTAAATCCTCATCAAAGGTGACGTTGGCCATGTTGGCCATG |
| Sp1-4_10_HCR_P2B2 | GGCCACATTGGCCACCGTGGCGACGAAATCATCCAgTAAACCgCC |

>araucan (XM_024082035.2_B1_AF546
TCTCGCGCGCCAGTCTGTGCTGAGATTTCGGAGTGCGAGGACGTCGTGCGAGCATGCTCACTCGGGAG
GACCTCTGACGCGCGATGGGCGGGCGCGCGCCGCCGCCGCGCACTGGACCGACTGTGATACATTAACT
TAACTAATTAGTTGTAATAAACCTTAGTATTAAACACGTAGTTCGCCGTCCGTGTCAGACTTTGAGAGA
CAGTGTGTGCCGTGAAGCCGCCCCTATGATCGTCCCTATGGCAGCGTATGCACAGTTCGGCTACTCCTA
CCCCTCCGCCTCGCAGTTGCTGGTGGGCAGCAGCGGCGGCGGGAACGGGGGAGCGGCGACGTCGCCC
GACGGCGGGTCCAGCTCCGGCCCGCCGCTGTCGCCCGGCGGGTCGGGGGTCGCTGGCGGCGCACTGTC
ACCGGGGGCCGGCTCGCACGCCAGCACCCCCGCCGCGCCGTGCTGCGACACCCCGCGCCCCATCATCA
CCGACCCCGTGTCCGGCCAGACGGTGTGCTCGTGCCAGTACGACGCCCGGCTAGCGCTCTCATCGTAC
CCGCGCTTGTCCAGCGCCGCGGTCGGCGTCTACGGAGCGCCGTACCCGTCCACGGATCAGAACCCTTA
CCCCAGCATCGGGGTTGACAGTTCGGCGTTCTACTCGCCTCTGAGCAACCCGTACGCTCTGAAAGAGG
GAAACGGCGAGATGTCAGCTTGGACATCGGCGGGCCTTCAACCCTCCGGCGGCTACTACCCCTACGAC
CCCACGCTCGCGGCCTACGGATACGGAGCGGGGTACGATCTAGCAGCCAGGCGGAAAAACGCGACGC
GCGAGTCCACCGCAACACTAAAGGCGTGGCTCAACGAGCACAAGAAGAACCCGTACCCGACCAAGGG
GGAGAAGATAATGTTGGCCATCATCACCAAGATGACGCTGACGCAGGTGTCCACGTGGTTCGCGAACG
CGCGCCGGCGGCTCAAGAAGGAGAACAAGATGACCTGGGAGCCCAAGAACAAGACAGACGACGACG
ACGACACCATGCTGTCCGACGAGGAGAAAGACGACGACAAATTAAAACCAAACAAAGATGAGGAAA
GGAAGGGGGACGAGCTTCTCCAGGGCATGCACAGTCAACTACTCGGCTACGGGATAAAGGAGGAGTC
CAAGCGAGGGACTTCAGACTGCGGCGTGCCGATACCAGCGTCGAAGCCCAAGATTTGGTCTTTAGCAG
ACACCGCGGCATGTAAAACGCCGCCACCGGCCGCGCAGCCGTGGCCTCAGCATGGATACGGGCCAGG
GCCGGAGCGGTCGGTGTCTGACGGTGGTGCAAACGGGTTCGCACTACCTGCGACGGCGGCCGCGAGTC
CAGCGAGCGGGTCGTATGGGAGATACGGCGGTTTCCCCGGACAGTACAACCAGCATCCGTGCGTGCAT
CCAGCGGCGTTCCCCGACGTTCAGACTGACACTCCGCCACAAACTCCGCCCAACATGAAGGTGCCCAG
CGTGGCCAACCCGCTGGGCAGCGGCGGCGGCTCCGGCTATTGTTTCCCTCGGCACCAGCAGTCGCCAC
AGCGGGACCCCTACCACAACCCTCACCATGCGAACAACCATCAACCCAACCAGCACCACAACGAGGG
TTCGGCCGCCTTTAAACCGTTCTACAAAAGGTAAAGCAAGTGGGTCCTGGGTTGCGGCACCTTCAACT
AGGAACTTCTAGAGACTTCTGAGAGATCACATTGGCGTAGTGTTTCACTCATCGAGTCAAGAAAATTG
GAGGAAGGGCGAGCGAGTCATCCTAGGGCTATACGCACATCGCCTAAATTTCAATTTCACTGACATGA
CAATCGTGACGTGGAGCCGAGATCGTTGCAGTGACATTTAAATCGTGTCGTGACGTCAAAATTTTAAT
GCAATTACGGTTTTAATGTCGTTGACAATGATTTAGTCTGGTTACAACATCTCTGGCTGATCTGGCCTG
CTGTCGCTCTAACATATGATGTATATTTAATTTCGTAATCTTTTGGTTATTTAGAATAATTTGAAATTCC
TAAATTTAAAAGCACAGACTATAAAAATGTAATATAATAAAAAAAAAGATCCATTAATTTGTACTTAAA
TGAGTATCATGTGATTCGTAGGCACTTACAGCTGTCGTCTTGGTACTTAAATCATGTATGGAAAAGTTG
CTCAGTATTTTTATAGTTATTAAGTATATTCGTTAAATAAATAACTATCCAAATTACTTATATACGACC
CAAACGCAATTAATAATTAATTATGTGTTAAATTAAACAATTAAAATAGCCATTGGTTTTAGTGAATTT
ATTGTAAAGTAGCCTTTACCCGCGTCTTCACTTCATTAGAAATAAGGGCGTTTAATGCCTTAAGACACC
GTTACATTTTTGGAATGAAAAGTACTATCACAATTAATTACGTACCACAAACAACCAGCACTGACTCG
TAATAAATCAGCAGATACACTGACATAATGACATAGATATAGAACAAAACCTTAAGGTTTTACCATA

TGCTACTAGCGCCATCTAGTATTTAGTTTTAATGAAGTTGGATACTAAATACTAAAAGCACCTATACAG
AAAAAAGTTAATTAACATACACAATTTCATAAATAAGTACCTACTGTGATCAGTCGCTCGACTATTAG
CTGAATTTTACATAGCACAGGATGTTTCACAATCGTGGCTTAGTTGAAGTCTTATGTCAGCCACTGACG
ATCAAGTAATCTCACATGCCTGCAGCGCTAACGGCTTGACGCCCGCTAAAACTAATCGCGTGTTTGTC
GCGATAGGCATGACATCATGCCGAGTTTTATCGGACGTTTTACTTTTTTATTACTGTTTTTAATAAAAC
GATAGTTTGGATATCAATAAAAATGAATAACATCACCACAAATAGCAAAACAAGACACAGATTTTTAA
GAAACTCCACATATTTAGGAACCTGAATATGAATAGTTCCTCGGTTAAGAGGTAGGACTTAACACCGG
AAGGTCACGGGTTCGAACCCCAACCGTTGGAATTTCTTGAGTATTTACGACTAGTTGGTGGGGAAACG
GAACATTCAAAAGGTACTAACTTCTAGATGGCGTTATATAAAAGCACTACATTTACAAAATTTGTTTCT
TTTGATTTTTATACCGGAATATTTTTTCTGGTTCAGTTAGTACTAAACAAACAATTTTATTAAATGTATA
GATTTAATTTATATGATAATCAATAGATTATTGTATTATTATTATAGTTTACTGTGTATTTAGTTCTTCC
AAACAAAATCGAAGTGTAATAACTATTGATCCTTCCGGAACGAAGTGACCTAATACATTTTTCTATGA
AAATCGATTATTGTAAAACATTTTATGTTTTTAAATCGTCACGATTTTTATATCTTACATCTAAAGCGCC
TATATGTGCAAAAACTTGCTTCATTTCTATGTATAAAAGTACAGCTTTCTTATTGATCGTCAGTTTTTTT
TTCATATTATCGTGTAATTAATGCACCGTCATAATTATTCATTAGCTTTTTGGTTGACTATGTTATGACA
CTTCGGAAGATTGTTATAATTCGTAGTGTTTAGATGTAACTACGTACCGAGCGTGTATTTAATGCCTAA
AGGAGGGTCGAATGAAGCATGTAGTTATAATGGTAATAATTTATTATATAAAAAAAAACTTCTACTAA
TTGTTTAAAACCTAATCCCGACATCTCTGTTTCAAAACTATGATGCGATCTATGCGGCTGATATTCTTA
TGGAAGTTTTCTATGAACAAAATCCAGAAAACGCTGAATATAATAATTGTTTGAGCATTTTGAGACCTT
CTGTGGCGTAATTGTGTAAAGATTTACATCACGGTGAACCTATGGTCGATTATCAGCACAGGTAAATG
TAACATTTTATATTTTTCAACAAATCAAACCAGGTTTAGTTTGGTGCAGATTTGATCTTGGCTAGTTAC
CACCCTACCCGCCAAAGACTTACGCCATTTAACTTAGCGTTCCAGTACTGAGCCATTAAGAGGTATAC
ATCTAATAAATTTATAACCTTTTTAAGTTAGCATAATACTATCAGACTGCATTATTTGTCAACAAAACA
TAGTCTACGGTTTGGTTGTTGAATAAATTAAAAAGAAACATTTCAATTACGCCTTTATTCAAATGAAAT
AAGATCAAAAATTCAGCCAAAATTAATTCGAAGATCGCAGTGCAATAATGACGAGTTTTGGAATAGA
AAGGCAGGGAATAGGTCCCTCGTCGTTGCGGCCCCCATCTTGAGACCCGTCTTTGCCCTAACGTTTTAT
TTGTTAAGCCTTTATCAATAATATTCTTCCATTCAGTTATTGTTGTTGTCATTGTAAATAGGCGGAGATT
TGTAGATTTTCTATTATTTAGTTTCACATCGAGTGTACAAAGTAATTAACTACTAATTAAATAATTTCAT
TTCCAACGATGGAACGCGTCGATTATAAATATATTGTTTAAAAAGTATTATTAGAAATATACTGTAAAT
AAATTTATTCTGAATATTAACTATATTATTTTATTTTATAAATATTAATATCGATCGCAGAATCAGTTTA
CAAACGTATCGGTAAGTATTTGTGAATAAAAAAAAAAGTAAATATATTTCGATGTTAAATAAGTCACGT
GGGTATTCATAAGGCACGTTTGAAATATACAATTAGCAGATTAGCACACGAGTAACCTTTGATGTAAT
GTCAGCCACTGACGATCAAGTAATCTCACAATCTCAACGGCGTGACATCATGCCGATCGCCATAACGA
AAGTTTTGTGGCCGATTCGATACCAGTTTACTAAAAAATGCTTTGTATCTTCTGGGGATTTTCATATGA
ACATGCGTTTAAAACAAAGGTTGCTAGTGCGCTAATCTGCTAATCGCACTAAGTTTTCGTGTAAACTAA
AACCCGGATTAATATAATGCTTTATTTACATTTCATTAAATGCGATAATTGTCTATCATTAACATGAAA
TAGTAGAATCATTTTAAACATTATTTAATTTAATAATCATGTTTATACTATAATCTGTGGTTCACATTAA
AAGCACTACGAGATAGGAGTTAGTTCTAAGAATAGTCAATTTATGTAAAAAAGGCAGTAAAGTAATTT
GTTGCTATTGTTCCTACAAAATAGTACACTTTGAAAACGAATTATCTCTCATTTGAGTATTTAAATTTA
GTATGAATTTTGATGAAAAAAATATTCAAGCCTACTCCGAACATCAATGTTGCTTTAGAAATATTTTGC
GAAGATAAATATTGGATTAGGTCTTACAAAACAAAAAATAAGTTTGTGATTTTACTTACTATAGCATTT
CAAGTTGACATGCCTTGTCCTTTGTTTATGACGTTTGTGTGAGAGATGCGATAACCCGAGTTACTCATA
GATTCCGTGGCTATCTCAAACGGTAATAAAAATAGAAATCCATCTTTATGTTAATAAAAAAATCGGTT
GTCTGTAAAGACGGTTTACTGATGACAGTAAAAAGTGACAACACCAGAAAATACTGATGGAATGGTG
CATTTTTGAAAAGAAAATTTTAACTAACGTATTTTACATAATAAATAAAATACTGTTCGTTTTTCTAAA
ATATTGTTTAATAATCTTCATAGACCAGAATATATTTTATTTCTTGTAAAAAAAGTTGGCAACCCTACA
GCGAGGGAACGACGCATGACGTCATCTTTTTTCGAGTGTGCAGGCTCCATCGAATTACAAGACGTTGT
CACGTCAAAAAAATACTGTTATGTGTGTGATTTAGTAAGTTTAATTGTAAACAGTAAACATGGTAACG
GCGCGTTTGAGAGGCACTTTATTAGGACGTAGCAACAGGGTTGTCAAGCTTTATGGACCTCGTTACAG
TAAAACTGGGGTTGAATTAAGTAATTCAGTAGTTTACTTATTCTTAGATTGTCATTTCCCTTATAGCAG
GGATGGCCAACAGGTCGATCGCGACTGTTAGTCCAGTAGATCGTCGCAAGGCAAAAAATATAAATAC
GTAGACCAGACTGCACAACGTAATCACCAACTCCTTCACGCATTATCTCGTATATTTTTTATAAAAATT
ATAATATATGATAGTTGACATAGAGAAGAAAAAAATTTCAACAATTTGTAAATATCAAGGTTCATTTAT
GAAAAAACGTCTACCTACCTACAACTTCATTTTTGAGAAGTAAACGACAGAAATTAGGGCAGTGGTAG
CAATCTATTGAACCAACTTCTCAAGTCGCTTTAGCGATTTGAGAAGTTGGTTCAATTCATCGGTTTAAT
GATTCCATTGATCAAAGTTATATCTATGAATTGGCTACTAAAGACGCAATAAGTACTCACATTATGTAA
TTGGTAGATCGTACAGGGTTTCAATAGTAAACAGGAGATCTTGGGTCTAATCAAGTTGGCCACCCCTG

CCTTATAGCATGAAGAGTTAAGAATGGAAATAAAACTGCTATTACAGCTGTATTACTTTACAGTGTAA
TGTGAGTCCTAAGGGTACCGCAACAAAACGCAGTAACTACAATTTATAACAATTTTTATATTTCTGTTA
TAAATAGTATGTACTCAGTATTGTTACTTTTTTTAATTCTGTCTTTACTGTTAATTATTTCATTTCTGTAA
AAATACATACATGGTTTACAGAAATTAAATAGGTTGATGGCTTAACGCTCAGGATTCTGACTGATGTA
CGACTCAAAATATTTTATTCAATTTAATTAGAATCTGATTAATTGAAGTGTGCACCTGTGCAATATCTA
AAGTTATAAAACAGTTTGATTTTTTTTTCCTTTTTTCGTCAAAAATTGTTATTAATTTTTTTTAACGTTTC
ACCTGTGTATTTTTTTACCCTTATTATGTTTCAAATCACCATTTCTACGAGTATAAAGGCAACGCTACA
AGGTGCAATATGATATCGAAATTACTACAGAGATATAGAAATACATTTTCTTCAAATACATTAAAATC
TGTATGAAAATTAAAAAAAAAGTGAGCTGTCCGGAGTTTAGTATGACTGTGAATTCTGAAACAAATAT
ACCGTAAAACTATAAGGCTAGCAAATGAGAGAGTTATTTTTTTAACTCGTTATTTATGTATCTATTTGC
TAACAAAAATAAAAAATGAAAATTAGAAAATGAATATTGAAGAGGGATACTAATTTATAGAGTCATA
AAAAGCCCGCAACGAGTTTTGTGTTCTAATCATAATATTAAATTACGAAATGTCAGTTTCATTCTTTTG
ACCCCCGATTTTTGCGTTAAAATGAAAATAGTCACGAGCTCAAAATGAATGAATTATATTGGACTGCA
TTGACTTGCAGGTTCACAAGCGTCCATACCCTTTTCAAATATAAAACGTATATTTAAAATATTTTCCTT
AGTATAAAACAAGAACTTATCATTGCGGGAAAGTAAAAACCTGTATTAGCGAAGGAATTTTCCATTTC
TTACAATTTGCAAGTCTAGTGCCTACCCTTAATAAAAACCTTATGCACGCCACCAATGGTATGGAGTAT
GTCTGGCTATCGCAAGCTCTTTAAGAAACTAGGACCCAGAATGTCATAACTTGTCTTATTAAACATAAT
TGTATATTCTTAGCTGTCACATGTAAAATCCCTTTATAGCTAATTGTTTTATAGTCACCCATAACATTTG
AGGCTTATATGCATTGACCAAACACGACATTGATTATACACGACCAAAGATTTCCTCTTATTTTAAAAG
ACTTCGTCGGTAAAATTTGATATTTTATCTATAATATCCAGCAATCGATATGTGGAAAAGGGTTTTGAA
CTGTGTATATTTGGTTGTCTATATCAATGATTGTTTGGTATGAAAATAGATTTTTTCAAAAAATAAACA
TAACAAACTTCAATGTGACCAAATCGAATACATTCGCTTAATTAATATTATAAAATGTTCTATACAGAT
ATTTATAGAATATGTACCTATTAATTATTATTATAAGTAAAATTATGTAACGATTGTAATTAAATCTTT
ATTTCAAAA

| | |
|---|---|
| ara1_P1B1 | gAggAgggCAgCAAACggAATCCCCCTTCCTTTCCTCATCTTTTA |
| ara2_P1B1 | gAggAgggCAgCAAACggAATGAAGTCCCTCGCTTGGACTCCTCC |
| ara3_P1B1 | gAggAgggCAgCAAACggAAGGTGGCGGCGTTTTACATGCCGCGG |
| ara4_P1B1 | gAggAgggCAgCAAACggAACGAACCCGTTTGCACCACCGTCAGA |
| ara5_P1B1 | gAggAgggCAgCAAACggAACTGGTTGTACTGTCCGGGGAAACCG |
| ara6_P1B1 | gAggAgggCAgCAAACggAAGGCACCTTCATGTTGGGCGGAGTTT |
| ara7_P1B1 | gAggAgggCAgCAAACggAAGGTCCCGCTGTGGCGACTGCTGGTG |
| ara1_P2B1 | TGACTGTGCATGCCCTGGAGAAGCTTAgAAgAgTCTTCCTTTACg |
| ara2_P2B1 | CGACGCTGGTATCGGCACGCCGCAGTAgAAgAgTCTTCCTTTACg |
| ara3_P2B1 | CCATGCTGAGGCCACGGCTGCGCGGTAgAAgAgTCTTCCTTTACg |
| ara4_P2B1 | GACTCGCGGCCGCCGTCGCAGGTAGTAgAAgAgTCTTCCTTTACg |
| ara5_P2B1 | GAACGCCGCTGGATGCACGCACGGATAgAAgAgTCTTCCTTTACg |
| ara6_P2B1 | CCGCTGCCCAGCGGGTTGGCCACGCTAgAAgAgTCTTCCTTTACg |
| ara7_P2B1 | TGTTCGCATGGTGAGGGTTGTGGTATAgAAgAgTCTTCCTTTACg |

>pannier (XM_052891510.1_B 2_AF647
CGACCGTGCGCCAGTCCGCGATCTGACCTCTGACCGCGGAGACGTGTCCGCGAGCGCGTCTGACCTCC
CGCGAGATAGCTGACCTCTCTGTGCCTCCGGCGCCTCGACATGGACTGCGGCATTCCTCAGGCGTGGC
TGCAGAACATTGCGCTGCTAGCAGGCAGCAGCGGCGTGGGCAGCGTGGGCGCCGTGGGCGGCGTGGG
CGGTGTGGGCGGCGTGGGCGGCGGGCTGTACGGCCAGAACATGGTCATGGGCTCGTGGTGCGCGCCCT
ACGACGCGCTGCAGCGCCCGCCGGCGTACGATGGCGTGCTGGACGCGTACGAGGAGGGCCGCGAGTG
CGTGAACTGCGGCGCCAACAACACGCCGCTGTGGCGCCGCGACTCCACGGGCCACTACCTGTGCAACG
CGTGCGGCCTGTACCACAAGATCAACGGCGTCAACCGCCCGCTCGTGAAGCCGAGCAAGCGGCTGTCG
GCGGCGCGGCGGCACGGCCAGTGCTGCACCAACTGCGGCTCGCGCAACACCACGCTGTGGCGCCGCA

ACAACGACGGCGAGCCGGTGTGCAACGCCTGCGGCCTGTACTACAAGCTGCACGGCATCAACAGGCC
TTTAGCGATGCGAAAAGATGGAATACAAACACGGAAACGAAAGCCGAAAAAGTCAGGGAACGGCGC
CAAGCCGCCGTCAGATGGCGCCAAGAAAGACGGACTCAACTCGCCGGGAATTGACGAAAGCAAGCCC
AGCATAGCAGAAGTGCCGCTGCCGCTGACAGCCGCCATGGCGGGCGCGGGCGGCAAGGCGCGCGAGC
CCGCGCCGTCCCCGCACGCGCACGCGCACGGGCACGGGCACGCGCACGCTCACGCGCACGCGCACGC
GCAGAGCACGCAGTACGCCGGGCACGCGCTGCCGCTGCCGAGCGCGCCCGCCTTCCTGTCCAACCCGT
CGCTGTTCAGCATCAAGAGCGAGCCCAGCGCGCCGGCCGGCTACGACGGCTACGCGCACTCCGGCTCC
GGCGCGCCCTACCACTCGCAGCAGCACTACCTGCATGCACTTCAGTACGGTCTGAGCAACGGCGACGA
GGAGGAGGGCAGCGGGTTCCTGCACCAGCGCAACGTGACGGCGCACGCCAAGCTCATGGCGTCCACG
TAGCAGCGCCGCCTCGCGCCGCGTGCTCGCCAGAGTTAGCGCACCGCTTGCGGCCCGCCGGGTCACCG
CCACGGGTCTCGTTCTGGAGTCCATGCTAAAACATATCCAGGACTGCTGCTTCCACAGATTGTGGCATA
TCCGTGATAGGTACAAGTACCTACCTACTTATGGAGATCTCATGGCTATCATAGTCAAAGCCTGCAGA
ATCAAACCATACCTACAGCCAGGAGTCATCGATTTTGCAAGAAATCTAAAGATTGAAATTTTTTTGA
AGAGACCTAATTAAATAGTAAGGACGGAGTGGCTGTCCCTAGGGATCGTGGAAGGTTGATGTACTTAC
CTAAGACTAAACACCGCCTGAAAGGATTAAAATCCGCCAAGTGCCTTGTTCAAATTCTTAGATAAGTG
CCTTACTTTTTAGTTCAAATTCTGTACGATGTAGGTATCTAAGTAGGTAAGACTGAAAACCACAGAGTT
TTTCTTACCTATATACCTACCAAACCATTTCTAGGGTACTACCTACCTACGAATTGTACCTACACTTAAT
TCTAAGCCATTGAGACTTACTTACTTATGTAATAAAAAATGTTCATTTGCTCTAGTCCGATGCAAATAT
TTTCTTGTTACACTGGCTTAGATACCCACGACTATTGTGATCAAGTAACAAGTACTGAGGTTTCTGTCA
GAATGTTTGTACGGCGATAAACGATTGTATATTTGTGTTAGATAAGGTTTATAATATGGAGATCAGTAT
TATTGGACTCGTTAATAAGGACCAGTCGTTCGCGAGACAACGCCAAGTCTGTTATTTCCGTGTCAGCCG
ACAGGTCTCGACTCTATGTAATTTGTGTAAAATATAAATTAGCGTAACACATTGAAGGTAGTGGAGCG
ATAAGGCGAATGTAGAAAGTAAAGTACCCACTTAGCGTGTAAATATCTAATTAAACAATTTCGACTGA
ACGATATGTAGTCACCGATGTGTAGGTGCCTACTTCGATGATAAGTTAAACTTGATCTGACTCGACGA
AGCGAGTTATTCTAATAAGTACTTAGATACATCACTAATGGCCTTATTGTTTAGCGACTTGATAACTAA
ATGCAAATTCGAACCTCTCGACTATTAATTAAATAGAGCCAAGTAATGACTGCACGTTCCGCTTATCTA
GTGTCGTACTCGTACTACCAGTGAACATAACTGGCGGTGAATCTGAGATACACTTCGCTCATTCGGAG
TGAAGGACTTGTACAGTTTATGACCAGTGTAGTTTGTTGAAAGTCCAGGACTGTTCGTACTTTTTAGAG
ATTCGTAGTTCATGCTTCTATTATTGTAGATAATGAAACCCTTTACAAAATTTTATTCTATAAACATACC
TACTCTACTGGTAAATACCTATTCACACTTATGTAGGACTAGCAGAACATCATGATTTCACCCACGTAG
TTCTCATTCCTTTAGGAATACGGGGATAAAGTGTATCTTATGTTACTCCCTGATAACGTAGCTTTCTATT
GGTGAAAGAATTTTTCAAATTGGTCCTTACGTAATAAAATACAAACCTTTCCTTTTTATAATATAGGTA
TTGATGTTGATATATAAGAAGCATGTTTTGTTTGAACTACGGGCTCCTACCTCATATGTATTCTGAACG
CTTGTCCAGTCATTTTAGTTATTAGTCAAATAAAGACGTATTAGTGTCCAATCATTGTTTATATACAAT
CGGCCTAGACTAGACTAGACCTATAGTACACTGTTGTAAAATACATAATTATAATAAAATTGTTAGTTT
CACATCTGCACATGTTTGGCCTCTCGGCGTAGCAATCTTATGAAAAAATAGATCTAAGAATAAATCGC
ATTTATTAATAGTTGTATATAGTAAAATTAATTAGCGAAATAATGTAAAGTCCGGAGTGCATATTCTGA
ACACTTTATTTAAAAACAACTTTTGATAACAACATACAAATGGCTCTGTGAAGAGAAATGTACAATAT
ATAATTAAACGTATGATGCACTA

| | |
|---|---|
| pnr1_P1B2 | CCTCgTAAATCCTCATCAAACGCCGCGCCGGTGTCAGTGGCGAGC |
| pnr2_P1B2 | CCTCgTAAATCCTCATCAAAAGTTGGTGCAGCACTGGCGTGCCGC |
| pnr3_P1B2 | CCTCgTAAATCCTCATCAAATTCCCTGACTTTTTCGGCTTTCGTT |
| pnr4_P1B2 | CCTCgTAAATCCTCATCAAACTATGCTGGGCTTGCTCTCGTCAAT |
| pnr5_P1B2 | CCTCgTAAATCCTCATCAAAGCTCAGACCGTACTGAAGTGCATGC |
| pnr1_P2B2 | GCCGCAGTTGGTGCAGCACTGGCGTAAATCATCCAgTAAACCgCC |
| pnr2_P2B2 | GCCACAGCGTGGTGTTGCGCGAGCCAAATCATCCAgTAAACCgCC |
| pnr3_P2B2 | GCGCCATCTGACGGCGGCTTGGCGCAAATCATCCAgTAAACCgCC |

| | |
|---|---|
| pnr4_P2B2 | CGGCCGTCAGCGGCAGCGGCACTTCAAATCATCCAgTAAACCgCC |
| pnr5_P2B2 | CCCGCTGCCCTCCTCCTCGTCGCCGAAATCATCCAgTAAACCgCC |

>drumstick (XM_024081055.2)_B3_AF488
GCGGAGCGGGGCGCGGGGCGCGTTCGGAAGCGGTGGCAGCTCCCATCGACTCCTGGGGTTATTCTTCC
TCAGGCCACTGCTGCTCGAGCTACGTACAGAGCGAGCCAGTTTTTTCATTCCACCGCACCGCACGCCA
CACACTCGTTAAGCCCGTGCCAACACGAGAAGTCGCCACTAATAAATTACTGTTTTTACTGCACTCGA
ACAGTCTACGCCGCGCAGCCGACTAATTTATACGTAAAAATAAACATCAATCAGCAATAAACGCCGCC
GTTAGCGCCCGAGCCCCGACACTGCGCACTAGCCCCGCCCGCAGCGAGCACCATAGCCCAGACAAAA
TGTTCGCCGTGATGCAAATCGACGAGGATCACACCAGGGACTTCAGGCGTAAGCTGCGCCCCAAGTGC
GAGTTTGTCTGCAAGTATTGCCAACGTCGGTTCACCAAGTCCTACAACCTGATGATACACGAGCGCAC
CCACAAGAGCCCCGAGCTGAGCTTCAGCTGCGAGGTCTGTGGGAAGAGCTTCAAAAGGCAGGACAAT
TTGCGTCAGCACAGATGTAGTCAATGCTTGTGGCGGTGAGGTGATGAAGAAGCACGATCAACAACAG
CGGTCTCTCTTACACAATAGAAGAAATACACTACAATTCCAAAAAGGAGACCACAAACATAAGATGG
TGATTCACTATGCAATCAGTCGGAGACCTGTATGTAGGTAAAAAACCACCTGGGAATTTTAACAGTAT
TCTTGCTTCTATTGTTGTACACCAGAATGGGATATTGCAATAACATTAAAAAAACTTCCGTCCCTTAAA
CATAAGGTATATTATATGTAAAAAATAATGTTAAAAATGATACTCTATCATGTGTCACTCTTAACATTT
TTTTAATAAATTATAATAATATCTACTTGATTAATTAAAA

| | |
|---|---|
| drm1_P1B3 | gTCCCTgCCTCTATATCTTTCTGCGGGCGGGGCTAGTGCGCGTTT |
| drm2_P1B3 | gTCCCTgCCTCTATATCTTTCCTGAAGTCCCTGGTGTGATCCTCG |
| drm3_P1B3 | gTCCCTgCCTCTATATCTTTGTAGGACTTGGTGAACCGACGTTGG |
| drm4_P1B3 | gTCCCTgCCTCTATATCTTTTCCCACAGACCTCGCAGCTGAAGCT |
| drm1_P2B3 | AACATTTTGTCTGGGCTATGGTGCTTTCCACTCAACTTTAACCCg |
| drm2_P2B3 | AAACTCGCACTTGGGGCGCAGCTTATTCCACTCAACTTTAACCCg |
| drm3_P2B3 | GTGGGTGCGCTCGTGTATCATCAGGTTCCACTCAACTTTAACCCg |
| drm4_P2B3 | GCAAATTGTCCTGCCTTTTGAAGCTTTCCACTCAACTTTAACCCg |

## REFERENCES AND NOTES


1. G. A. Boxshall, The evolution of arthropod limbs. *Biol. Rev. Camb. Philos. Soc.* **79**, 253–300 (2004).

2. G. E. Budd, The morphology of Opabinia regalis and the reconstruction of the arthropod stem-group. *Lethaia* **29**, 1–14 (1996).

3. J. F. Coulcher, "Evolution of the Arthropod Mandible: A molecular developmental perspective," thesis, University College London (2011).

4. J. F. Coulcher, M. J. Telford, Cap'n'collar differentiates the mandible from the maxilla in the beetle Tribolium castaneum. *Evodevo* **3**, 25 (2012).

5. R. E. Snodgrass, *In Principles of Insect Morphology* (McGraw-Hill, 1935), p. 256.

6. L. M. Nagy, M. Grbic, Cell lineages in larval development and evolution of holometabolous insects, *The Origin and Evolution of Larval Forms*, 275–300 (1999).

7. J. Bitsch, The controversial origin of the abdominal appendage-like processes in immature insects: Are they true segmental appendages or secondary outgrowths? (Arthropoda Hexapoda). *J. Morphol.* **273**, 919–931 (2012).

8. R. E. Snodgrass, Part I. General structure of the abdomen and its appendages, in *Morphology of the insect abdomen* (Smithsonian Miscellaneous Collections, 1931), vol. 85 pp. 1–128.

9. H. E. Hinton, On the structure, function and distribution of the prolegs of the Panorpoidea, with a criticism of the Berlese-Imms theory. *Ecol. Entomol.* **106**, 455–540 (2009).

10. S. J. R. Birket-Smith, Prolegs, legs and wings of insects, *Entomonograph*, vol. 5, p. 128 (1984).

11. R. W. Warren, L. Nagy, J. Selegue, J. Gates, S. Carroll, Evolution of homeotic gene regulation and function in flies and butterflies. *Nature* **372**, 458–461 (1994).

12. Y. Suzuki, M. F. Palopoli, Evolution of insect abdominal appendages: Are prolegs homologous or convergent traits? *Dev. Genes Evol.* **211**, 486–492 (2001).

13. H. S. Bruce, N. H. Patel, Cryptic persistence of truncated abdominal legs in insects enabled diverse outgrowths with novel functions (Preprints, 2023); https://doi.org/10.20944/preprints202212.0268.v3.

14. N. Suzuki, *Embryology of the Mecoptera (Panorpidae, Panorpodidae, Bittacidae and Boreidae)* (Bulletin of the Sugadaira Montane Research Center, Tsukuba, Japan: University of Tsukuba, 1990) vol. 11, pp. 1–87.

15. J. Tan, B.-Z. Hua, Morphology of immature stages of Bittacus choui (Mecoptera: Bittacidae) with notes on its biology. *J. Nat. Hist.* **42**, 2127–2142 (2008).

16. C. Yue, B. Hua, Are abdominal prolegs serially homologous with the thoracic legs in Panorpidae (Insecta: Mecoptera)? Embryological evidence. *J. Morphol.* **271**, 1366–1373 (2010).

17. R. Machida, External features of embryonic development of a jumping bristletail, Pedetontus unimaculatus Machida (Insecta, Thysanura, Machilidae). *J. Morphol.* **168**, 339–355 (1981).

18. Y. Ikeda, R. Machida, Embryogenesis of the dipluran *Lepidocampa weberi* Oudemans (Hexapoda, Diplura, Campodeidae): External morphology. *J. Morphol.* **237**, 101–115 (1998).

19. H. S. Bruce, N. H. Patel, Knockout of crustacean leg patterning genes suggests that insect wings and body walls evolved from ancient leg segments. *Nat. Ecol. Evol.* **4**, 1703–1712 (2020).

20. G. Panganiban, A. Sebring, L. Nagy, S. Carroll, The development of crustacean limbs and the evolution of arthropods. *Science* **270**, 1363–1366 (1995).

21. R. Janssen, B. J. Eriksson, G. E. Budd, M. Akam, N.-M. Prpic, Gene expression patterns in an onychophoran reveal that regionalization predates limb segmentation in pan-arthropods. *Evol. Dev.* **12**, 363–372 (2010).

22. C. Estella, R. S. Mann, Non-redundant selector and growth-promoting functions of two sister genes, *buttonhead* and *Sp1*, in *Drosophila* leg development. *PLOS Genet.* **6**, e1001001 (2010).

23. N. D. Schaeper, N.-M. Prpic, E. A. Wimmer, A clustered set of three Sp-family genes is ancestral in the Metazoa: Evidence from sequence analysis, protein domain structure, developmental expression patterns and chromosomal location. *BMC Evol. Biol.* **10**, 88 (2010).

24. S. N. Murugesan, H. Connahs, Y. Matsuoka, M. D. Gupta, G. J. L. Tiong, M. Huq, V. Gowri, S. Monroe, K. D. Deem, T. Werner, Y. Tomoyasu, A. Monteiro, Butterfly eyespots evolved via cooption of an ancestral gene-regulatory network that also patterns antennae, legs, and wings, *Proc. Natl. Acad. Sci. U.S.A.* **119**, e2108661119 (2022).

25. Z. Zheng, A. Khoo, D. Fambrough Jr., L. Garza, R. Booker, Homeotic gene expression in the wild-type and a homeotic mutant of the moth *Manduca sexta*, *Dev. Genes Evol.* **209**, 460–72 (1999).

26. D. L. Lewis, M. DeCamillis, R. L. Bennett, Distinct roles of the homeotic genes *Ubx* and *abd-A* in beetle embryonic abdominal appendage development. *Proc. Natl. Acad. Sci. U.S.A.* **97**, 4504–4509 (2000).

27. M. Masumoto, T. Yaginuma, T. Niimi, Functional analysis of *Ultrabithorax* in the silkworm, *Bombyx mori*, using RNAi. *Dev. Genes Evol.* **219**, 437–444 (2009).

28. H. Xiang, M. W. Li, J. H. Guo, J. H. Jiang, Y.P. Huang, Influence of RNAi knockdown for E-complex genes on the silkworm proleg development. *Arch. Insect Biochem. Physiol.* **76**, 1–11 (2011).

29. B. Konopová, E. Buchberger, A. Crisp, Transcriptomics supports that pleuropodia of insect embryos function in degradation of serosal cuticle to enable hatching. bioRxiv 584029 [**Preprint**]. 22 March 2019. https://doi.org/10.1101/584029.

30. G. Vachon, B. Cohen, C. Pfeifle, M. E. McGuffin, J. Botas, S. M. Cohen, Homeotic genes of the Bithorax complex repress limb development in the abdomen of the *Drosophila* embryo through the target gene *Distal-less*. *Cell* **71**, 437–450 (1992).

31. K. Ueno, C. C. Hui, M. Fukuta, Y. Suzuki, Molecular analysis of the deletion mutants in the E homeotic complex of the silkworm *Bombyx mori*. *Development* **114**, 555–63 (1992).

32. S. Tomita, A. Kikuchi, *Abd-B* suppresses lepidopteran proleg development in posterior abdomen. *Dev. Biol.* **328**, 403–409 (2009).

33. M.-H. Pan, X.-Y. Wang, C.-L. Chai, C.-D. Zhang, C. Lu, Z.-H. Xiang, Identification and function of Abdominal-A in the silkworm, Bombyx mori. *Insect Mol. Biol.* **18**, 155–160 (2009).

34. X. L. Tong, M. Y. Fu, P. Chen, L. Chen, Z. H. Xiang, C. Lu, F. Y. Dai, Ultrabithorax and abdominal-A specify the abdominal appendage in a dosage-dependent manner in silkworm, Bombyx mori. *Heredity* **118**, 578–584 (2017).

35. H. Wang, X. Tong, M. Liu, H. Hu, Z. Li, Z. Xiang, F. Dai, C. Lu, Fine Mapping of a Degenerated Abdominal Legs Mutant (Edl) in Silkworm, Bombyx mori. *PLOS ONE* **12**, e0169224 (2017).

36. A. Monteiro, Distinguishing serial homologs from novel traits: Experimental limitations and ideas for improvements. *Bioessays* **43**, e2000162 (2021).

37. H. Connahs, S. Tlili, J. van Creij, T. Y. J. Loo, T. Banerjee, T. E. Saunders, A. Monteiro, Distal-less activates butterfly eyespots consistent with a reaction diffusion process. *Development* **146**, dev169367 (2019).

38. N. Yoshiyama, K. Tojo, M. Hatakeyama, A survey of the effectiveness of non-cell autonomous RNAi throughout development in the sawfly, *Athalia rosae* (Hymenoptera). *J. Insect Physiol.* **59**, 400–407 (2013).

39. A. Beermann, M. Aranda, R. Schröder, The *Sp8* zinc-finger transcription factor is involved in allometric growth of the limbs in the beetle *Tribolium castaneum. Development* **131**, 733–742 (2004).

40. N. D. Schaeper, N. M. Prpic, E. A. Wimmer, A conserved function of the zinc finger transcription factor Sp8/9 in allometric appendage growth in the milkweed bug *Oncopeltus fasciatus*. *Dev. Genes Evol.* **219**, 427–435 (2009).

41. T. Königsmann, N. Turetzek, M. Pechmann, N.-M. Prpic, Expression and function of the zinc finger transcription factor Sp6-9 in the spider Parasteatoda tepidariorum. *Dev. Genes Evol.* **227**, 389–400 (2017).

42. H. Jeon, J. O, S. Jin, J. Lim, C. P. Choe, A Role for *buttonhead* in the Early Head and Trunk Development in the Beetle *Tribolium castaneum*. *Dev. Reprod.* **23**, 63–72 (2019).

43. S. Thümecke, R. Schröder. The *odd-skipped* related gene *drumstick* is required for leg development in the beetle *Tribolium castaneum*. *Dev. Dyn.* **251**, 1456–1471 (2022).

44. H. S. Bruce, How to align arthropod legs. bioRxiv 427514 [**Preprint**]. 13 November 2021. https://doi.org/10.1101/2021.01.20.427514.

45. M. Abu-Shaar, R. S. Mann, Generation of multiple antagonistic domains along the proximodistal axis during *Drosophila* leg development. *Development* **125**, 3821–3830 (1998).

46. N.-M. Prpic, R. Janssen, B. Wigand, M. Klingler, W. G. M. Damen, Gene expression in spider appendages reveals reversal of exd/hth spatial specificity, altered leg gap gene dynamics, and suggests divergent distal morphogen signaling. *Dev. Biol.* **264**, 119–140 (2003).

47. A. M. Cheatle Jarvela, C. S. Trelstad, L. Pick, Regulatory gene function handoff allows essential gene loss in mosquitoes. *Commun. Biol.* **30**, 540 (2020).

48. M. Holzem, N. Braak, O. Brattström, A. P. McGregor, C. J. Breuker, Wnt Gene Expression During Early Embryogenesis in the Nymphalid Butterfly *Bicyclus anynana*. *Front. Ecol. Evol.*, **7**, 468 (2019).

49. T. Ohde, T. Yaginuma, T. Niimi, Insect Morphological Diversification Through the Modification of Wing Serial Homologs. *Science* **340**, 495–498 (2013).

50. I. Almudi, J. Vizueta, C. D. R. Wyatt, A. de Mendoza, F. Marlétaz, P. N. Firbas, R. Feuda, G. Masiero, P. Medina, A. Alcaina-Caro, F. Cruz, J. Gómez-Garrido, M. Gut, T. S. Alioto, C. Vargas-Chavez, K. Davie, B. Misof, J. González, S. Aerts, R. Lister, J. Paps, J. Rozas, A. Sánchez-Gracia, M. Irimia, I. Maeso, F. Casares, Genomic adaptations to aquatic and aerial life in mayflies and the origin of insect wings. *Nat. Commun.* **11**, 2631 (2020).

51. Y. Hu, D. M. Linz, A. P. Moczek, Beetle horns evolved from wing serial homologs. *Science* **366**, 1004–1007 (2019).

52. C. R. Fisher, J. L. Wegrzyn, E. L. Jockusch, Co-option of wing-patterning genes underlies the evolution of the treehopper helmet. *Nat. Ecol. Evol.* **4**, 250–260 (2020).

53. Y. Matsuoka, A. Monteiro, Melanin Pathway Genes Regulate Color and Morphology of Butterfly Wing Scales. *Cell Rep.* **24**, 56–65 (2018).

54. H. M. T. Choi, M. Schwarzkopf, M. E. Fornace, A. Acharya, G. Artavanis, J. Stegmaier, A. Cunha, N. A. Pierce, Third-generation *in situ* hybridization chain reaction: Multiplexed, quantitative, sensitive, versatile, robust. *Development* **145**, dev165753 (2018).

55. H. S. Bruce, G. Jerz, S. Kelly, J. McCarthy, A. Pomerantz, G. Senevirathne, A. Sherrard, D. A. Sun, C. Wolff, N. H. Patel, Hybridization Chain Reaction (HCR) In Situ Protocol, protocols.io. (2021); https://dx.doi.org/10.17504/protocols.io.bunznvf6.

56. B. Bushnell, BBMap: A Fast, accurate, splice-aware aligner; https://sourceforge.net/projects/bbmap/ [accessed 19 November 2020].

57. D. Kim, J.M. Paggi, C. Park, C. Bennett, S. L. Salzberg. Graph-based genome alignment and genotyping with HISAT2 and HISAT-genotype. *Nat. Biotechnol.* **37**, 907–915 (2019).

58. M. Pertea, D. Kim, G. M. Pertea, J. T. Leek, S. L. Salzberg, Transcript-level expression analysis of RNA-seq experiments with HISAT, StringTie and Ballgown. *Nat. Protoc.* **11**, 1650–1667 (2016).

59. A. J. Hart, S. Ginzburg, M. S. Xu, C. R. Fisher, N. Rahmatpour, J. B. Mitton, R. Paul, J. L. Wegrzyn, EnTAP: Bringing faster and smarter functional annotation to non-model eukaryotic transcriptomes. *Mol. Ecol. Resour.* **20**, 591–604 (2020).

60. B. J. Haas, A. Papanicolaou, M. Yassour, M. Grabherr, P. D. Blood, J. Bowden, M. B. Couger, D. Eccles, B. Li, M. Lieber, M. D. MacManes, M. Ott, J. Orvis, N. Pochet, F. Strozzi, N. Weeks, R. Westerman, T. William, C. N. Dewey, R. Henschel, R. D. LeDuc, N. Friedman, A. Regev, De novo transcript sequence reconstruction from RNA-seq using the Trinity platform for Reference generation and analysis. *Nat. Protoc.* **8**, 1494–1512 (2013).

61. M. I. Love, W. Huber, S. Anders, Moderated estimation of fold change and dispersion for RNA-seq data with DESeq2. *Genome Biol.* **15**, 550 (2014).

62. G. Struhl, A homoeotic mutation transforming leg to antenna in *Drosophila*. *Nature* **292**, 635–638 (1981).

63. S. W. Herke, N. V. Serio, B. T. Rogers, Functional analyses of *tiptop* and *antennapedia* in the embryonic development of *Oncopeltus fasciatus* suggests an evolutionary pathway from ground state to insect legs. *Development* **132**, 27–34 (2005).

64. P. Chen, X. L. Tong, D. D. Li, M. Y. Fu, S. Z. He, H. Hu, Z. H. Xiang, C. Lu, F. Y. Dai, *Antennapedia* is involved in the development of thoracic legs and segmentation in the silkworm, Bombyx mori. *Heredity* **111**, 182–188 (2013).

65. T. Nagata, Y. Suzuki, K. Ueno, H. Kokubo, X. Xu, C.-C. Hui, W. Hara, M. Fukuta, Developmental expression of the *Bombyx Antennapedia* homologue and homeotic changes in the *Nc* mutant. *Genes Cells* **1**, 555–568 (1996).
